# There Is (Scientific) Strength in Numbers: A Comprehensive Quantitation of Fc Gamma Receptor Numbers on Human and Murine Peripheral Blood Leukocytes

**DOI:** 10.3389/fimmu.2020.00118

**Published:** 2020-02-05

**Authors:** Christina Kerntke, Falk Nimmerjahn, Markus Biburger

**Affiliations:** ^1^Division of Genetics, Department of Biology, Friedrich-Alexander University Erlangen-Nürnberg, Erlangen, Germany; ^2^Medical Immunology Campus Erlangen, Friedrich-Alexander University Erlangen-Nürnberg, Erlangen, Germany

**Keywords:** Fc receptors, antibodies, human leukocytes, murine leukocytes, quantification, receptor numbers, neutrophils, monocytes

## Abstract

Antibodies are essential mediators of immunological defense mechanisms, are clinically used as therapeutic agents, but are also functionally involved in various immune-mediated disorders. Whereas IgG antibodies accomplish some of their biological tasks autonomously, many functions depend on their binding to activating and inhibitory Fcγ receptors (FcγR). From a qualitative point of view expression patterns of FcγR on immunologically relevant cell types are well-characterized both for mice and humans. Surprisingly, however, there is only quite limited information available on actual quantities of FcγR expressed by the different leukocyte populations. In this study we provide a comprehensive data set assessing quantitatively how many individual human and mouse FcγRs are expressed on B cells, NK cells, eosinophils, neutrophils, basophils and both classical, and non-classical monocytes under steady state conditions. Moreover, among human donors we found two groups with different expression levels of the inhibitory FcγRIIb on monocytes which appears to correlate with haplotypes of the activating FcγRIIIa.

## Introduction

Antibodies of the immunoglobulin G (IgG) isotype are essential mediators of immunological defense mechanisms. Whereas they carry out some of their biological functions autonomously, such as blocking of cell surface receptors, most of their functions depend on the binding of the antibody Fc-domain to specific receptors, the Fc gamma receptors (FcγR). Whereas binding of immune complexes (IC) is possible to all FcγR including the low/medium receptors FcγRIIa and FcγRIIb, FcγRIIIa and FcγRIIIb in man and FcγRIIb, FcγRIII and FcγRIV in mice, monomeric IgG molecules can only bind to human or murine high affinity receptor FcγRI [reviewed e.g., in (1–3)].

The repertoire of murine and human FcγR includes the inhibitory FcγRIIb, which contains an intracellular immunomodulatory tyrosine based inhibitory motif (ITIM) whereas most other FcγR carry an activating ITAM motif. These motifs are either located in the same polypeptide chain as the ligand binding domain (FcγRIIb and FcγRIIIb) or on an accessory Fc receptor gamma chain. One exception is the human FcγRIIIb which contains no transmembrane or intracellular polypeptide domain, but is rather linked to the cell membrane by a GPI anchor (4).

Immune cell populations are characterized by a typical expression pattern of FcγR. Whereas most of them express both activating and inhibitory FcγR, B cells only express the inhibitory FcγRIIb, which regulates the activity of the B cell receptor. Natural Killer (NK) cells on the other side selectively express the activating FcγRIII. Thus, from a qualitative point of view expression patterns of FcγR on different immune cell subsets are well-characterized both, for mice and man (1–3, 5–12).

For many cell types, their functional response to external signals is accompanied by pronounced up- or down-regulation of various surface receptors. This clearly shows that the amount of any given surface receptor on a cell is an important characteristic for its physiological functionality. The outcome of immune complex binding to a cell is determined by the sum of activating and inhibitory signals triggered through the respective activating and inhibitory FcγRs. One factor influencing these signals is the affinity of the respective IgG isotype to the distinct FcγRs present on the cell surface. This led to the development of the concept of the so called A/I ratio as a prediction of the outcome of binding of IgG molecules of a given isotype based on its affinities to the inhibitory and activating receptors, respectively (13–15). Based on the A/I ratio concept mathematical models have been developed predicting IgG activity (16). However, it is to be expected that other factors such as the avidity of the immune complex which is influenced by the number of immunoglobulins bound to the antigen and the number of activating and inhibitory receptors on the surface of the respective effector cell may influence IgG effector functions (17). Thus, knowledge of the quantities of the various FcγR on the cell surface of immune cell subsets may help to develop more precise models to understand how IgG antibodies trigger cellular responses.

Whereas FcγR expression is rather well-described qualitatively, there is only quite limited information available on actual numbers of FcγR expressed by the different leukocyte populations (18–35). To our knowledge there is no publication, where such numbers have been determined for the main leukocyte populations in parallel, even though Antal-Szalmas et al. (18) and the group of Guyre (19, 22–25) provided data on human neutrophils and monocytes. In addition, work on FcγR cell surface numbers often dates back to times, where certain cell subpopulations with an entirely different FcγR repertoire (such as classical and non-classical monocytes), had not been distinguished.

Flow cytometry is a common method for the analysis of cell surface receptors. Qualitative comparison of the relative expression of a given cell surface receptor on different cell populations based on the fluorescence intensities mediated by binding of fluorochrome-labeled antibodies specific for this receptor is rather straightforward. In contrast, quantitative comparison of different receptors on the same or different cell populations by simple comparison of fluorescence intensities is not possible since there are inherent variations between the different anti-receptor antibody conjugates with respect to their specific fluorescence (i.e., fluorescence intensity per molecule). This is obvious when conjugates with different fluorochromes are used. However, even antibodies conjugated with the same fluorochrome may differ in the stoichiometry of fluorophores per antibody molecule. This variability can be partially reduced by using conjugates with very large fluorophores like R-Phycoerythrin (PE), where usually not more than one fluorochrome can be linked to each antibody molecule (36). In spite of this advantage of PE studies by David et al. suggest that there is also a variation in the quantum yield of PE from various organisms (37). The information, from which species the used PE is derived from, is usually not available from the antibody companies or providers of labeling kits. In addition, also for PE the stoichiometry of fluorophores per antibody molecule for any lot of antibody conjugate may be <1 due to partial degradation or incomplete coupling.

To achieve a more definitive estimate of receptor numbers per cell the establishment of reference curves comparing the observed fluorescence to the fluorescence of well-defined reference beads appears to be better suited. Thus, we used sets of commercially available beads with distinct numbers of binding sites for anti-FcγR antibodies, respectively, and established reference curves for all anti-FcR monoclonal antibody (mAb) conjugates used in this study. Based on these references we present here the ABC values (i.e., the *a*ntibody-*b*inding *c*apacity for respective anti-FcγR antibodies) for all activating and inhibitory FcγR on the main leukocyte populations in peripheral blood of C57BL/6 and FcγR knockout mice and in healthy human volunteers under steady state conditions. This includes Natural Killer (NK) cells, B cells, T cells, neutrophils, eosinophils and basophils as well as classical and non-classical monocytes. These monocyte subsets have comparable population size in murine blood. In contrast, in humans the classical monocytes represent the vast majority of monocytes whereas non-classical monocytes represent only a small fraction of the monocyte population but nonetheless exert important biological functions. In both species these subsets have differential FcγR repertoires and, thus, specific effector functions upon engagement with immune complexes [see e.g., (38)].

## Materials and Methods

### Mice

Female mice at 8–16 weeks of age on C57BL/6 background were used in all experiments. C57BL/6J mice (JAX strain 000664) were purchased from Janvier (Le Genest-Saint-Isle, France). FcγRI deficient mice (39) were originally provided by M. Hogarth (JAX number: not available (N/A)), FcγRIIb deficient [(40), JAX number: N/A], FcγRIII deficient (JAX number N/A) and FcγRIV deficient [(41), JAX number: N/A] mice by J. Ravetch. Mice were kept in the animal facilities of Friedrich-Alexander-University Erlangen-Nürnberg under specific pathogen-free conditions in individually ventilated cages according to the guidelines of the National Institutes of Health and the legal requirements in Germany. Animal experiments conducted in the animal facility of the FAU were approved by government of lower Franconia.

### Human Donors

For the characterization of human leukocytes venous blood of male and female healthy adults was used. The use of human material for scientific purposes was carried out in accordance with the recommendations of and approved by the ethics committee of the Friedrich-Alexander University Erlangen-Nürnberg. All subjects gave written informed consent regarding usage of their biological material for the scientific research presented here.

### Preparation of Murine and Human Peripheral Blood Leukocytes

For isolation of murine PBLs blood was drawn from the retro-orbital plexus using anti-coagulant micro hematocrit capillaries. Human PBLs were isolated from venous blood of male and female healthy human adults using anticoagulant EDTA Monovettes. Erythrocytes from both murine and human blood were lysed using deionized H_2_O and subsequent restoration of iso-osmolality. After repeated washing in cold FACS buffer containing sodium azide to inhibit changes in the surface presentation of proteins, cells were continued processing for flow-cytometric analysis.

### Flow Cytometry

#### Characterization of Murine PBLs

Single cell suspensions with typically 1–2 × 10^5^ cells per sample were usually incubated for 15 min on ice with Fc-block antibodies to minimize unspecific binding to Fc receptors, followed by staining with fluorochrome-coupled antibodies for ~20 min. Since single FcγRs were to be stained specifically, full Fc block was inconvenient. We, thus, pretreated cells with anti-CD16/32 clone 2.4G2 to block FcγRII and III only when FcγRIV was quantified. This Fc block was not used in analyses of FcγRI, since 2.4G2 may also block high affinity receptor FcγRI via its Fc-part on cells were the antibody is bound in cis to FcγRII or III (42). Since we have recently shown that also medium-affinity receptor FcγRIV can bind the Fc-part of several rat and mouse IgG subclasses and cause false positive results in flow cytometry (43), FcγRIV was blocked by clone 9E9 in all analyses where other receptors than FcγRIV were to be analyzed.

For the identification of cell populations we used antibodies against the following antigens: B220 (clone RA3-6B2, APC conjugated, BD Biosciences or FITC conjugated Biolegend), CD3e (clone 145-2C11, FITC conjugated, Biosciences and Biolegend or BV510 or AlexaFluor 647 conjugated, Biolegend), CD11b (clone M1/70, PerCP-Cy5.5 conjugated), CD19 (clone 6D5, BV510 conjugated, Biolegend), CD45 (clone 30-F11, APC-Cy7 conjugated BD Biosciences and Biolegend or APC-Fire750 conjugated, Biolegend), CD49b (clone DX5, APC conjugated, BD Biosciences), CD62L (clone MEL-14, PE-Cy7 conjugated, Biolegend), Gr1 (clone RB6-8C5, APC- or AlexaFluor 647 conjugated, BD Biosciences and Biolegend or BV510 conjugated, Biolegend), IgE (clone R35-72, FITC conjugated, BD Biosciences), Ly6G (clone 1A8, FITC-conjugated, provided by BD Biosciences and Biolegend), NK1.1 (clone PK136, FITC conjugated, Southern Biotech, BD Biosciences and Biolegend), and TCRß (clone H57-597, FITC conjugated, Biolegend). Flow cytometric measurements were carried out on a FACSCanto II (BD Bioscience). Briefly, cell aggregates were excluded by their light scatter characteristics. Dead cells were excluded from analysis using 4′,6- diamidino-2-phenylindole (DAPI). Single viable CD45^+^ leukocytes were divided into SSC^high^ granulocytes and further distinguished into neutrophils and eosinophils by Ly6G. Among SSC^low^ cells NK cells were characterized as NK1.1^+^ CD11b^intermediate^ and monocytes as NK1.1^−^ CD11b^high^. Monocytes were further distinguished by expression of CD62L and Gr1 (high for classical monocytes and negative-low for non-classical monocytes). Among SSC^low^ CD11b negative cell B cells were characterized by binding of anti-B220 whereas T cells were B220 negative and, in addition, in some experiments were characterized as positive for TCRß or CD3e. Murine basophils were identified as cells with low side-scatter characteristics which were negative for lineage markers CD19, CD3 and Gr-1 but were CD49b^+^ and positively stained for IgE. A representative gating for murine leukocytes is shown in Supplementary Figure 1.

#### Characterization of Human PBLs

Antibodies detecting the following antigens were used for the characterization of human leukocyte populations: CD3 (clone SK7, PE-Cy conjugated, BD Biosciences), CD14 (clone HCD14, FITC conjugated and clone M5E2, PerCP-Cy5.5 conjugated, Biolegend), CD16 (clone 3G8, PE-Cy7 conjugated, BD Biosciences), CD19 (clone HIB19, APC conjugated, BD Biosciences and Biolegend), CD33 (clone WM53, BV510 conjugated, Biolegend), CD45 (clone HI30, APC-Fire750 or APC-H7 conjugated, Biolegend), CD56 (clone MEM188, PerCP-Cy5.5 conjugated and FITC conjugated, Biolegend), CD123 (clone 6H6, PE-Cy7 conjugated, Biolegend), FcεRIA (clone AER-37 (CRA-1), PerCP-Cy5.5 conjugated, Biolegend), HLA-DR (clone L243, APC conjugated, Biolegend). Per sample typically 1–2 × 10^5^ human leukocytes were used.

To avoid unspecific binding of antibodies for the quantification of human Fc receptors to any other FcγR via their Fc part, we utilized the Human TruStain FcX^™^ Fc Receptor Blocking Solution (Biolegend). This Fc-block protects from Fc-mediated binding to FcγRs by pre-occupying their Fc binding sites, but does not inhibit antigen-specific detection of anti-huFcγR antibodies which takes place with higher affinity. In pretests we verified that this reagent did not affect antigen-specific FcγR-detection, prior to its employment in quantification experiments (Supplementary Figure 2). FcγRIIb quantification took additional advantage from the fact that the anti-FcγRIIb antibody is a 2B6-variant where N-glycosylation is prevented. Since binding to FcγRs via the Fc-part is impaired for a-glycosylated antibodies (44, 45), undesirable Fc-mediated binding of 2B6 to other Fc receptors is prevented. Flow cytometric measurements were carried out on a FACSCanto II (BD Bioscience). Briefly, cell aggregates were excluded by their light scatter characteristics and dead cells were excluded from analysis by DAPI staining.

Among CD45^+^ leukocytes, neutrophils and eosinophils were identified by their high granularity resulting in their distinct light scatter characteristics (SSC^high^) and distinguished by the CD16 expression of neutrophils and/or by the intrinsic auto-fluorescence of the eosinophils. Among SCC^low^ cells NK cells were gated as being CD56 positive but negative for CD14 and CD33. Monocytes were identified in the CD56^−^ population by the expression of CD33 and CD14. They were further distinguished as classical CD14^high^ CD16^low^ and much less frequent non-classical CD14^low^ CD16^+^ monocytes. Within the CD33- CD14- population B cells were identified by their expression of CD19 and T cells by the expression of CD3 in absence of CD56. Basophils were identified as SSC^low^ CD45^dim^ CD123^+^ HLA-DR negative cells, which were positively stained for IgE receptor FcεR1. In addition to leukocytes we analyzed human platelets, which were characterized by their small size as reflected by low light scatter and by their expression of CD41a. A representative gating strategy for human leukocytes and platelets is shown in Supplementary Figure 3.

### Quantification of Fc Receptors

Fcγ receptors on leukocytes were quantified by measuring their Antibody Binding Capacity (ABC) for antibodies specific for the respective FcγR. ABC values on leukocytes were calculated using a specific reference curve for the correlation between fluorescence intensity of a cell upon binding by the respective fluorochrome-conjugated anti-FcγR antibody and the number of antibody binding sites. These reference curves were generated using sets of Quantum Simply Cellular (QSC) microspheres (Bangs Laboratories Ltd.) with known numbers of antibody binding sites as provided by the manufacturer. Beads and cells were stained with the same concentration of the respective anti-FcγR antibodies. Reference curves were established in each experiment for the analyzed anti-FcγR antibodies. According to manufacturer's instructions a titration curve should be prepared for every quantitating antibody using the QSC beads to determine its saturating concentration. However, earlier experiments suggested that this concentration might not be sufficient to saturate all binding sites on cells for all antibodies. Insufficient saturation of binding sites on cells with full saturation on reference beads will lead to an underestimation of the number of binding sites on the target cells. The difference between saturating concentrations for cells and QSC beads is easily conceivable since binding to cells is achieved via antigen-specific binding domains in the Fab regions whereas binding to beads is achieved via other antibody domains probably via the Fc-region and binding to both entities may take place with very different binding affinities. We, thus, suggest titrating quantitating antibodies both for binding to cells and to QSC beads in order to aim at saturating binding to both entities.

The following PE-conjugated anti-FcγR antibodies were used for mouse receptor quantification: anti-msFcγRI/CD64 mouse IgG1 clone X54-5/7.1 (BD Biosciences), anti-msFcγRIIb/CD32b mouse IgG2a clone Ly17.2 (in-house production and labeling), anti-msFcγRIII/CD16 rat IgG2a clone 275003 (R&D Systems), anti-msFcγRIV Arm. hamster IgG clone 9E9 (in-house production and labeling or Biolegend).

PE-labeled antibodies specific for human FcγR were: anti-huFcγRI/CD64 mouse IgG1 clone 10.1 (BD Biosciences), anti-huFcγRIIb/CD32B humanized IgG1 clone 2B6 (in-house production and labeling) and anti-huFcγRIII/CD16 mouse IgG1 clone 3G8 (Biolegend). Since no fully FcγRIIa/CD32A-specific mAb was at hand, expression of this receptor was analyzed by staining with a anti-CD32 antibody (clone IV.3, antibodies-online GmbH) after pre-blocking of CD32B with clone 2B6 N297Q which recognizes specifically FcγRIIb but not FcγRIIa (46). Among anti-human FcγRII antibodies IV.3 appears to be one of the clones with the most preferential binding to FcγRIIa over FcγRIIb. However, under conditions of saturating binding to FcγRIIa expressing leukocytes, which are important for reliable quantification of antibody-binding sites by this method, even IV.3 reveals pronounced binding to FcγRIIb e.g., on B cells (Supplementary Figure 4). Binding of IV.3 to FcγRIIb can be efficiently blocked by pre-treatment with anti-FcγRIIb antibody 2B6 (10 μg/ml) without affecting binding to highly FcγRIIa-expressing monocytes (Supplementary Figure 4). Thus, pre-blocking of FcγRIIb is a versatile and necessary step for reliable quantification of FcγRIIa expression by IV.3.

Human FcγRs I and III could be detected directly by antibody clones 10.1 and 3G8, followed by using anti-mouse IgG QSC beads. In a similar manner, human FcγRIIb was detected with the antibody clone 2B6 and by using anti-human IgG QSC beads. To quantify FcγRIIb we used a PE-conjugated recombinant 2B6 whose Fc part is of human origin. Thus, we employed Quantum^™^ Simply Cellular^®^ (QSC) anti-human beads to establish a reference curve for 2B6 binding sites.

Anti-FcγR antibodies were purchased from BD Biosciences, BioLegend, R&D Systems Europe or prepared in house. To minimize potential systematic variations in the quantification of different receptors by engagement of different fluorophores we used a single type of fluorophore. We chose Phycoerythrin (PE) for this purpose, since due to its size there is typically one fluorochrome conjugated to each antibody molecule, thereby minimizing variations in specific fluorescence and it lacks the pronounced self-quenching capacity of fluorochromes like FITC (47). Anti-FcγR antibodies were either purchased pre-labeled or were conjugated in-house. According to the host species of the respective anti-FcγR antibody, we used anti-mouse IgG, anti-rat IgG or anti-human IgG QSC beads following manufacturer's instructions. Since anti-FcγRIV is derived from Armenian hamster and no QSC beads specifically binding antibodies of this species are available, we performed a sandwich-assay where anti-mouse IgG beads were pre-coated with mouse anti-hamster antibody. The formulation of the latter is a commercially available equal mixture (BD Biosciences) of two murine antibody clones specific for either hamster IgG1 or hamster IgG2-3, respectively. The anti-FcγRIV clone 9E9 is an Armenian hamster IgG not further characterized regarding the IgG subtype. It should, thus bind to one of these two mouse IgG clones. Assuming that upon loading QSC microspheres with this mixture half of the anti-mouse IgG binding sites on the microspheres are loaded with the antibody clone which binds 9E9 and each of these antibody molecules has two binding sites for 9E9 we assumed the capacity of the QSC microspheres for 9E9 binding to be equal to the mouse IgG binding capacity of these microspheres as provided by the manufacturer.

To enable subtraction of ABC-background values based on background fluorescence of the respective cells, we used FMO (“fluorescence-minus-one”) controls in each experiment, where cells were stained with all antibodies except the anti-FcγR antibody. Flow cytometric analysis was done on a FACS Canto II (BD Biosciences, Heidelberg). Data were analyzed with FACSDiva Software (BD). For ABC calculation we used QuickCal^®^ software provided by Bangs Laboratories.

An example for the quantification procedure from flow cytometric analysis to calculation of ABC values is provided in Supplementary Figure 5.

### Identification of Allelic Variants for Human Donors

For the identification of FcγR haplotypes of human donors, genomic DNA was isolated from peripheral blood and stored at −20°C. For genotyping of FcγRIIb^232I/T^ und FcγRIIb^G/C−386/A/T−120^ alleic variants a nested PCR was carried out with an initial long-range PCR (93°C 15″, 68°C 17′ for 10 cycles; 93°C 15″, 68°C 28′ for 28 cycles; PCR product ~17 kbp) using primers LR-FOR (5′ ctccacaggttactcgtttctaccttatcttac 3′ and LR-REVERSE (5′ gcttgcgtggcccctggttctca 3′) PCR products were extracted from Agarose gels and used as template for a second PCR, either using primer pair PP-FOR (5′ caatttaccgagagcaagacagc 3′) and PP-REVERSE (5′ gcagtcagcccagtcactctcagt 3′) for amplification of promoter polymorphism alleles (95°C 30″, 58° 30″, 72°C 90″ for 35 cycles; final elongation 72°C 5′; PCR product 1946 bp) or primer pair I232T-FOR (5′ cctgcctgctcacaaatgta 3′) and I232T-REVERSE (5′cactgctctccccaagac 3′) for amplification of alleles for the I232T transmembrane polymorphisms (98°C 15″, 58°C 20″, 72°C 30″ for 35 cycles; final elongation 72°C 7′; PCR product ~750 bp). Products of both PCRs were eluted from agarose gels and sequenced by GATC Biotech using primer PPseq (5′ tgacatacctccttgtccttgtt 3′) for the promoter polymorphism and I232T-FOR for the polymorphism in the transmembrane region.

Polymorphisms of FcγRIIa^131H/R^ and FcγRIIIa^158V/F^ were characterized by nested allele-specific PCR. For FcγRIIa^131H/R^ an initial PCR (94°C 5′, 56°C 5′, 72°C 5′ for 10 cycles, followed by 94°C 60″, 56°C 60″, 72°C 2′ for 30 cycles, final elongation 72°C, 10′) with primer pairs IIa-1st FORWARD (5′ ggagaaaccatcatgctgag 3′) and IIa-1st REVERSE (5′ gaagagctgcccatgctg 3′) was performed to amplify a *fcgr2a* specific DNA fragment containing the polymorphism. This was followed by an allele-specific PCR with common IIa131-REVERSE primer (5′ caattttgctgctatgggc 3′) and either FORWARD primer IIa131H (5′ gaaaatcccagaaatttttcca 3′) or IIa131R (5′ gaaaatcccagaaatttttccg 3′) which could only amplify the histidine or arginine variant, respectively. Successful PCR reactions provided a 249 bp fragment. For FcγRIIIa^158V/F^ an initial PCR (94°C 5′; followed by 94°C 60″, 58°C 30″, 72°C 2.5′ for 30 cycles, final elongation 72°C, 10′) with primer pairs IIIa-1st FORWARD (5′ gtgtctttcaggctggctg 3′) and IIIa-1st REVERSE (5′ gaccagaatagtttaatctcg 3′) was performed to amplify a *fcgr3a* specific DNA fragment containing the polymorphism. This was followed by an allele-specific PCR with common IIIa158-FORWARD primer (5′ tcacatatttacagaatggcaaagg 3′) and either REVERSE primer IIIa158V (5′ tctctgaagacacatttctactccctac 3′) or IIIa158F (5′ tctctgaagacacatttctactccctaa 3′) which could only amply the histidine or arginine variant, respectively. Successful PCR reactions provided a 138 bp fragment.

### Statistics

Panels with data sets for analysis of statistical significance are depicted as bar charts. Data in bar charts are expressed as mean + standard deviation if all data sets are normally distributed and as median ± interquartile range (IR) if at least one data set in the panel is not normally distributed according to Shapiro-Wilk test. Otherwise, sets data are provided either in box plots or as individual data points. Statistical significance of differences between medians of two sets of data was analyzed by Mann-Whitney test. Data were analyzed and plotted with Graph Pad Prism software (GraphPad Software Inc., San Diego, CA).

## Results

In the present work we analyzed the expression of Fcγ receptors I, IIb, III, and IV on murine peripheral blood leukocytes and Fcγ receptors I, IIa, IIb, and IIIa/b on human peripheral blood leukocytes by flow cytometric analysis with fluorochrome-conjugated antibodies against the various Fcγ receptors (anti-FcR). FcγR expression on cells is referred to as their antibody binding capacity (ABC, depicted as *antibody-binding sites per cell*) for the respective anti-FcR antibodies. To achieve this, fluorescence intensity of anti-FcR antibody bound cells and cells from FMO controls without anti-FcR antibody was translated into ABC, using reference curves that were established in each experiment for all tested anti-FcR antibodies using Quantum^™^ Simply Cellular^®^ (QSC) microspheres. Anti-mouse, anti-rat or anti-human QSC microspheres (Bangs Laboratories Ltd.) were used according to the host species of the respective anti-FcγR antibody.

### Quantification of Mouse Fcγ Receptors

**Figure 1:**
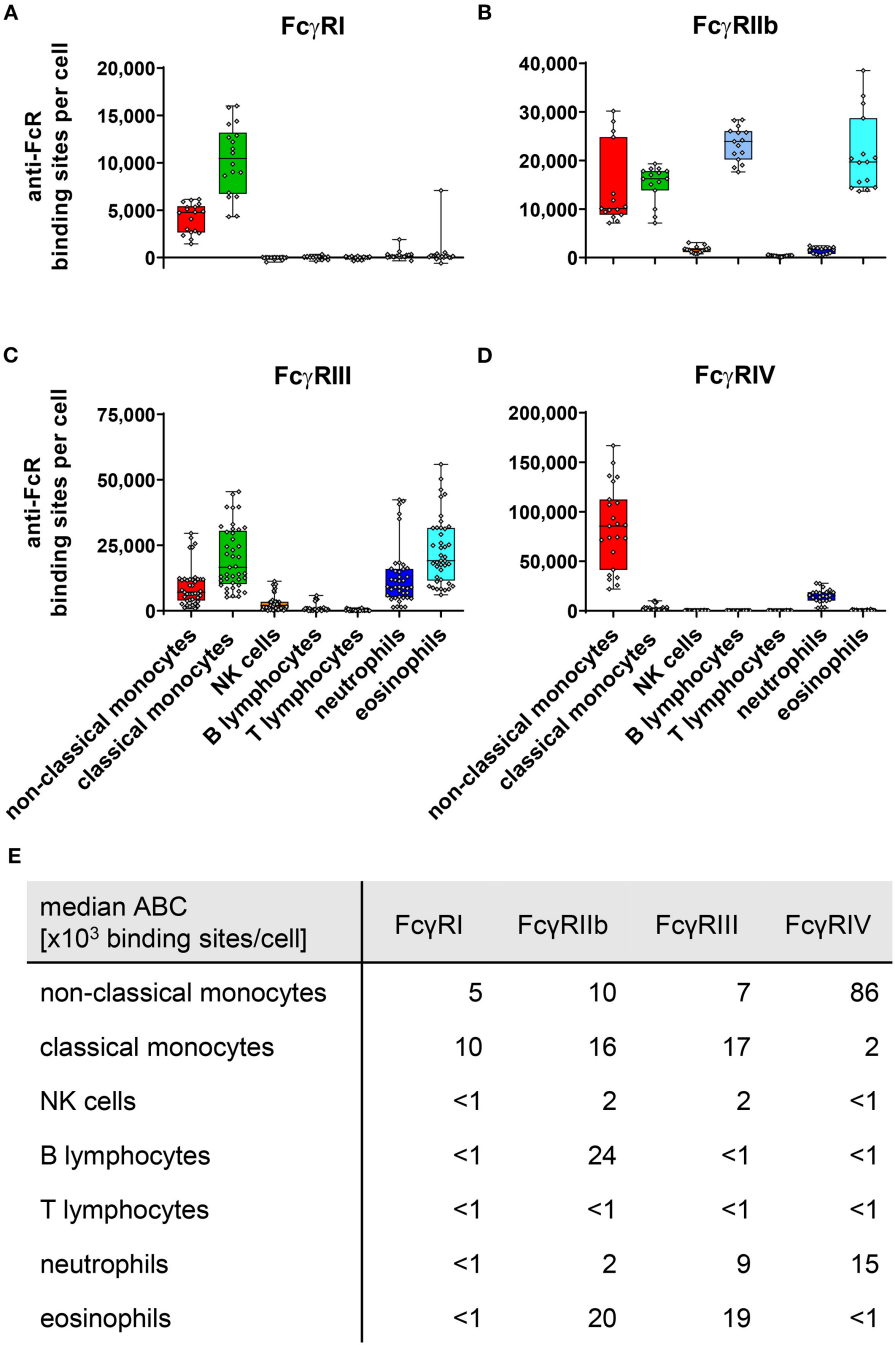
Fc gamma receptors on murine peripheral blood leukocytes. Depicted are box plots showing anti-FcR binding sites for **(A)** FcγRI, **(B)** FcγRIIb, **(C)** FcγRIII, and **(D)** FcγRIV on indicated leukocyte subsets together with **(E)** a tabular presentation of the median number of binding sites. *n* = 15–41 from 4 to 11 independent experiments.

Using this methodology, we quantified the ABC for murine Fcγ receptors I (CD64), IIb (CD32b), III (CD16), and IV using PE-labeled anti-FcγR antibodies on peripheral blood leukocytes of C57BL/6 mice under steady state conditions. Quantification of mouse FcγRs was done repeatedly over several years by different researchers and by using different lots of QSC beads, antibodies and antibody providers. The summary of these measurements are depicted in Figure 1. In contrast to merely qualitative analyses our quantitative approach also allows direct comparison of ABC values for activating vs. inhibitory receptors on the different cell types that co-express both receptors.

As shown in Figure 1, expression of the high affinity FcγRI was restricted to monocytes, with classical monocytes expression roughly twice as much FcγRI than non-classical monocytes. With 24,000 ABC per cell, the inhibitory FcγRIIb was expressed most strongly on B cells, closely followed by eosinophils with 20,000 ABCs. On eosinophils FcγRIIb expression levels were matched by a nearly similar expression of activating FcγRIII (1.9 × 10^4^ binding sites). Co-expression of inhibitory and activating receptors was also found on mouse monocytes. On classical monocytes FcγRIIb (1.6 × 10^4^ binding sites) expression levels faced a slightly higher number of activating receptors comprising mainly of FcγRIII (1.7 × 10^4^ binding sites) and FcγRI (1 × 10^4^ binding sites), but also some FcγRIV (2 × 10^3^ binding sites). This FcγRIV expression may be due to some classical monocytes upregulating FcγRIV in the process differentiation into non-classical monocytes (48, 49). For FcγRIV on non-classical monocytes, we calculated the highest expression of all murine FcγRs (8.6 × 10^4^ binding sites). In contrast to this high FcγRIV expression, moderate levels of activating FcγRs FcγRI (5 × 10^3^ binding sites) and FcγRIII (7 × 10^3^ binding sites) were noted. The inhibitory FcγRIIb was only expressed at intermediate levels on this monocyte subset (1 × 10^4^ binding sites) and remained about one order of magnitude below activating FcγR numbers.

Low values of anti-FcγRIIb binding sites per cell have been measured on NK cells and neutrophils (both 2 × 10^3^ binding sites), as well as FcγR-lacking T-cells (<1 × 10^3^ binding sites) upon staining with self-labeled anti-FcγRIIb-PE. However, in comparison to cell subsets known to express the inhibitory FcγRIIb, such as monocytes, eosinophils, and B-cells these values—especially for T cells—appear negligible. At this point, however, we cannot explicitly distinguish whether there is in fact very low FcγRIIb expression at least on NK cells and neutrophils, or whether for example some free PE molecules from the Ly-17.2 in-house labeling were capable of binding to the cells. Even if the low expression of the inhibitory FcγRIIb on neutrophils was indeed real under steady state conditions, it faces pronounced expression of activating FcγRIV (1.5 × 10^4^ binding sites) and moderate levels of FcγRIII (9 × 10^3^ binding sites) on these cells. Finally, we could verify our previously published observation that murine NK cells express only low levels of FcγRIII (50) (2 × 10^3^ binding sites) during the steady state.

### FcγR Expression by FcγR Knockout Mice

**Figure 2:**
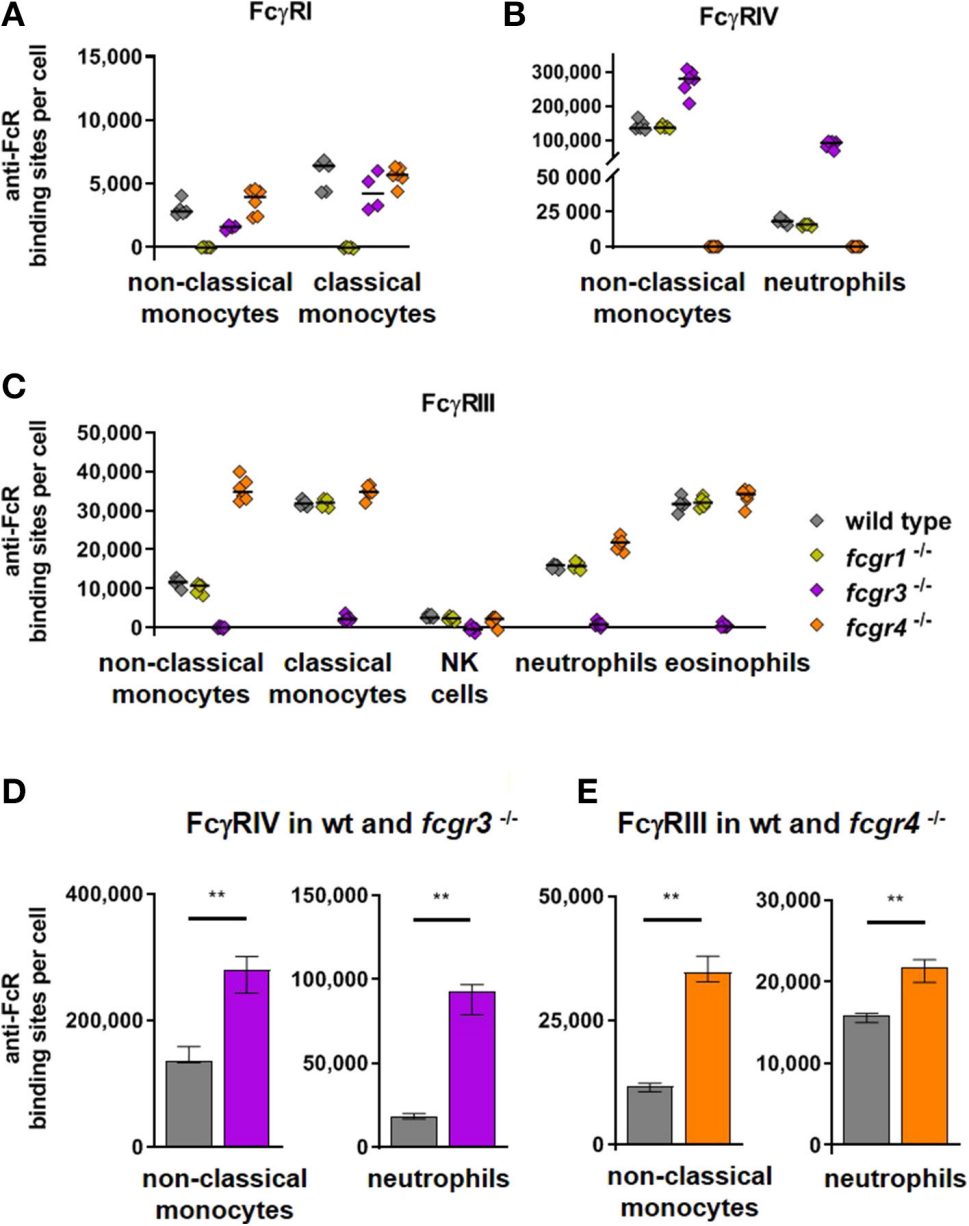
Activating Fc gamma receptors on PBLs of wild type and activating FcγR knockout mice. In **(A–C)** individual values of anti-FcγR binding sites on cell populations prominently expressing the respective receptors are depicted for C57BL/6 wt mice and knockout mice lacking FcγRI, FcγRIII, or FcγRIV. Data are shown **(A)** for FcγRI on monocytes, **(B)** for FcγRIV on non-classical monocytes and neutrophils and **(C)** for FcγRIII on monocytes, NK cells, neutrophils and eosinophils. In **(D–E)** the anti-FcγR binding sites per cell are show for groups with prominently increased activating receptors on knockout compared to wild type mice, i.e., in **(D)** for FcγRIV in *fcgr3*^−/−^ knockout mice (violet bars) and in **(E)** for FcγRIII in *fcgr4* ^−/−^ knockout mice (orange bars) in comparison to C57Bl/6 wild type mice (gray bars) on non-classical monocytes and neutrophils. *n* = 5–6; median ± IR; Mann-Whitney test for significance; **p < 0.01.

It has been reported previously that deficiencies in FcγR expression may modify the expression of other FcγRs. For example FcγRIV expression on neutrophils was increased in FcγRIII knockout mice (51–53), but not in FcγRI deficient mice (51, 53). In addition we had observed that deletion of FcγRIV resulted in a slight up-regulation of FcγRIII on neutrophils (52). These results were corroborated in the present work where we analyzed potential compensatory effects upon deficiency of single activating receptors in a parallel analysis of C57BL/6, FcγRI-, FcγRIII- and FcγRIV- knockout mice. Figure 2 depicts anti-FcR binding sites for FcγRI on monocytes (Figure 2A), and FcγRIV on non-classical monocytes and neutrophils (Figure 2B) (Supplementary Figure 6 for other cell types also) as well as FcγRIII on monocytes, NK cells, neutrophils and eosinophils (Figure 2C) for C57BL/6 mice and knockout mice lacking FcγRI, FcγRIII, or FcγRIV. This analysis revealed that FcγRIV expression was significantly increased by nearly a factor of two on non-classical monocytes of FcγRIII deficient mice (2.8 × 10^5^ antibody binding sites per cell) compared to C57BL/6 mice (1.4 × 10^5^ binding sites) (Figure 2D). On neutrophils FcγRIII deficiency increased FcγRIV expression even about 5-fold (9.3 × 10^4^ in FcγRIII deficient mice vs. 1.8 × 10^4^ in wt mice). Among FcγRIII expressing cells lack of FcγRIV resulted in a significant increase of FcγRIII on neutrophils (2.2 × 10^4^ in FcγRIV deficient mice vs. 1.6 × 10^4^ in wt mice) and 3-fold higher ABC values on non-classical monocytes (3.5 × 10^4^ in FcγRIV deficient mice vs. 1.2 × 10^4^ in wt mice) (Figure 2E).

**Figure 3:**
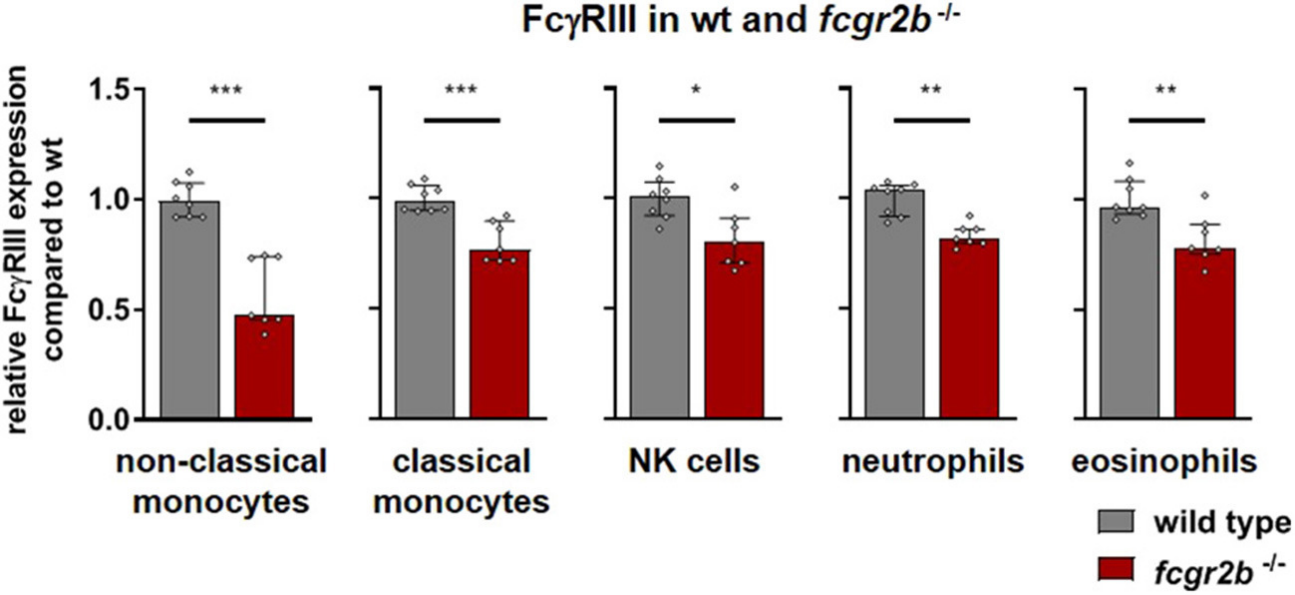
Activating Fc gamma receptors on PBLs of wild type and inhibitory FcγR knockout mice. Depicted are the anti-FcγRIII binding sites per cell for wild type (gray bars) and *fcgr2b*^−/−^ knockout mice (red bars) from two experiments, normalized to the mean expression in the respective wild type cohort. *n* = 7–8; Shown is the median ± IR together with the individual values; Mann-Whitney test for significance; *p < 0.05; **p < 0.01; ***p < 0.001.

In FcγRIIb deficient mice the activating FcγRIII was moderately down regulated compared to wild type mice on most cell subsets (Figure 3). On non-classical monocytes, however, FcγRIII expression was reduced by about one half according to the combined results of two independent experiments (Figure 3). FcγRI and FcγRIV appeared unaffected by the FcγRIIb knockout (Supplementary Figure 7).

### Quantification of Human Fcγ Receptors

**Figure 4:**
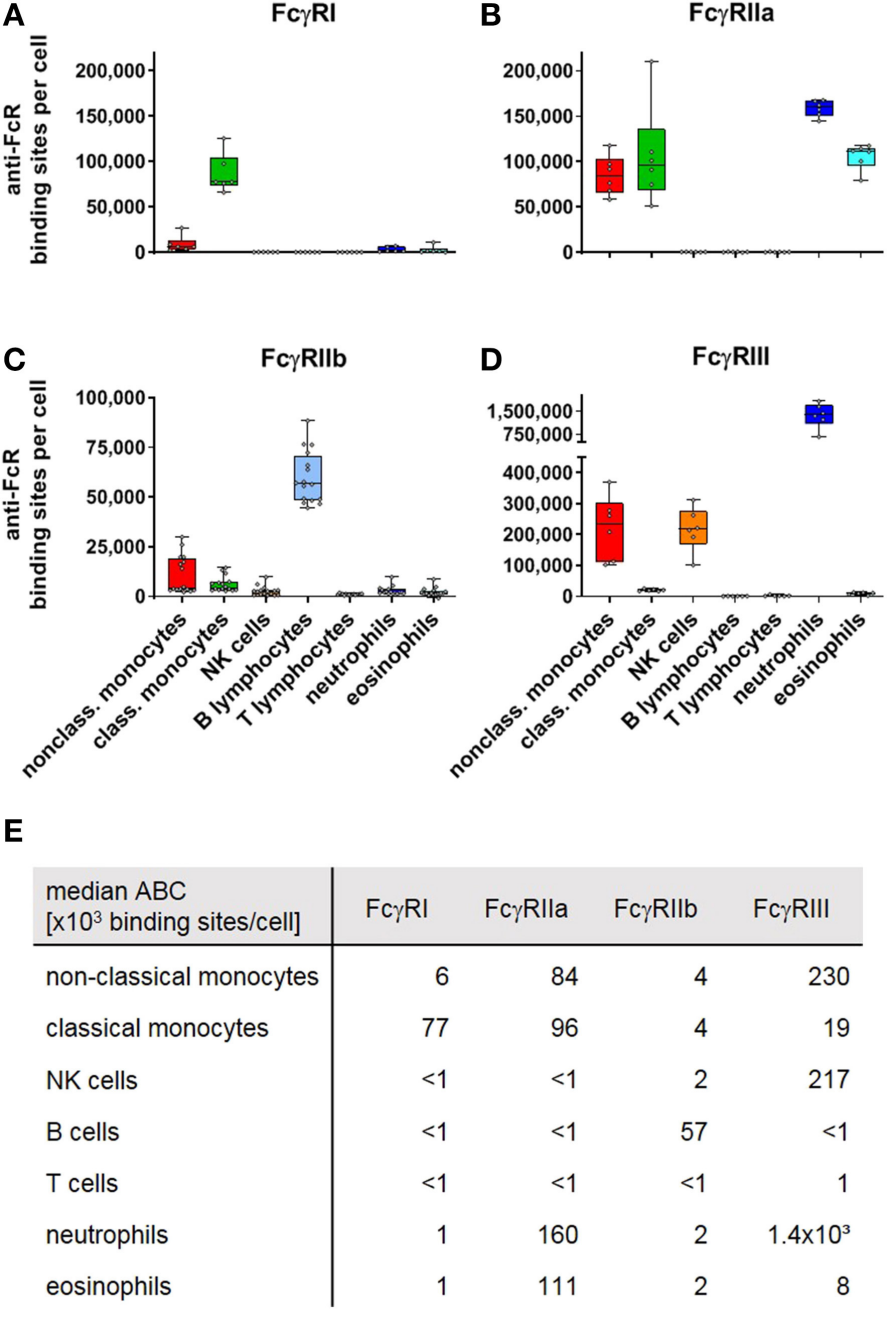
Fc gamma receptors on human peripheral blood leukocytes. Depicted are box plots showing anti-FcR binding sites for **(A)** FcγRI, **(B)** FcγRIIa, **(C)** FcγRIIb, and **(D)** FcγRIIIa/b on indicated leukocyte subsets together with **(E)** a tabular presentation of the median number of binding sites. *n* = 6 (FcγRI, IIa, III) and 16 (FcγRIIb).

In addition to quantifying mouse FcγR-expression, we also assessed human FcγR expression. Thus, we quantified expression levels for inhibitory FcγRIIb and the closely related but ITAM-bearing activating FcγRIIa on human leukocytes. We also studied but did not experimentally distinguish both variants of human FcγRIII, i.e., FcγRIIIa with a canonical polypeptide transmembrane domain and the GPI-anchored FcγRIIIb. As the latter receptor is exclusively expressed on neutrophils, and at low levels on basophils (54), however, this should not impact on the reported numbers. The calculated anti-FcR binding sites for the human Fc receptors are depicted in Figure 4. In the literature, FcγR expression has been mainly studied on human neutrophils and monocytes by various methods (18–34). According to our results, FcγRI is barely expressed by neutrophils in steady-state, consistent with observations by others (18–29). On monocytes, FcγRI expression was described with numbers ranging from about 1–4 × 10^4^ anti-FcγRI or monomeric IgG binding sites (18, 19, 22, 23, 27, 30–33), but in contrast to our work these studies did not distinguish between monocyte subsets. As shown in Figure 4A non-classical monocytes have low to moderate FcγRI expression (6 × 10^3^ anti-FcγRI binding sites). In contrast, classical monocytes (~8 × 10^4^ binding sites) expressed considerably higher numbers of FcγRI. The same was true for FcγRII where numbers between 2 and 4.7 × 10^4^ molecules per cell have been published (18, 19, 22, 23), without differentiating between activating and inhibitory FcγRII receptors or between classical and non-classical monocytes. In our study, we calculated for classical monocytes ~1 × 10^5^ binding sites for FcγRIIa and 4 × 10^3^ binding sites for FcγRIIb whereas non-classical monocytes had ABC values of ~8 × 10^4^ binding sites for FcγRIIa and 4 × 10^3^ binding sites for FcγRIIb. For neutrophils, where we calculated 1.6 × 10^5^ anti-FcγRIIa binding sites but barely any FcγRIIb expression (2 × 10^3^ binding sites), published pan-FcγRII values are in the range of ~1–4.5 × 10^4^ (18, 19, 22–25, 34). The ABC values for the inhibitory FcγRIIb were the highest on B cells (5.7 × 10^4^). For FcγRIIIa on NK cells we calculated 2.2 × 10^5^ binding sites whereas 4.4 × 10^4^ (22) and 7.9 × 10^4^ (35) receptor numbers were reported in other studies. Eosinophils had an ABC for FcγRIIIa of 8 × 10^3^ binding sites according to our data compared to a receptor number of 1.2 × 10^4^ reported by others (35). Our data on FcγRIIIa expression on non-classical monocytes revealed 2.3 × 10^5^ sites per cell whereas numbers of ~3.5 × 10^4^-1.2 × 10^5^ have been published by others (18, 19, 22). For neutrophils we extrapolated an ABC for FcγRIII (i.e., mainly FcγRIIIb) of 1.4 × 10^6^ binding sites per cell which is as well higher than the values provided by others (18, 19, 22–26, 34, 35) (~6 × 10^4^-4 × 10^5^) using antibody clones CLB-Gran/1, ION16, and mostly 3G8. As the anti-FcγRIII-PE fluorescence was beyond the fluorescence of the reference beads with respect to the highest number of antibody binding sites, the ABC values for neutrophils are being extrapolated by extending the calculated reference line (linear correlation in a log/log scheme) beyond the highest reference point. This reduces the accuracy of the calculation of anti-FcγRIII binding sites on human neutrophils, but in every experiment performed with human leukocytes so far the fluorescence of neutrophils upon anti-FcγRIII staining had been higher than that of the respectively used reference beads with the highest binding capacity. This was also true in an assay where we used one lot of beads with ~620,000 sites, the highest number of binding sites we noted so far.

### Differential Expression of FcγRIIb on Human Monocytes

**Figure 5:**
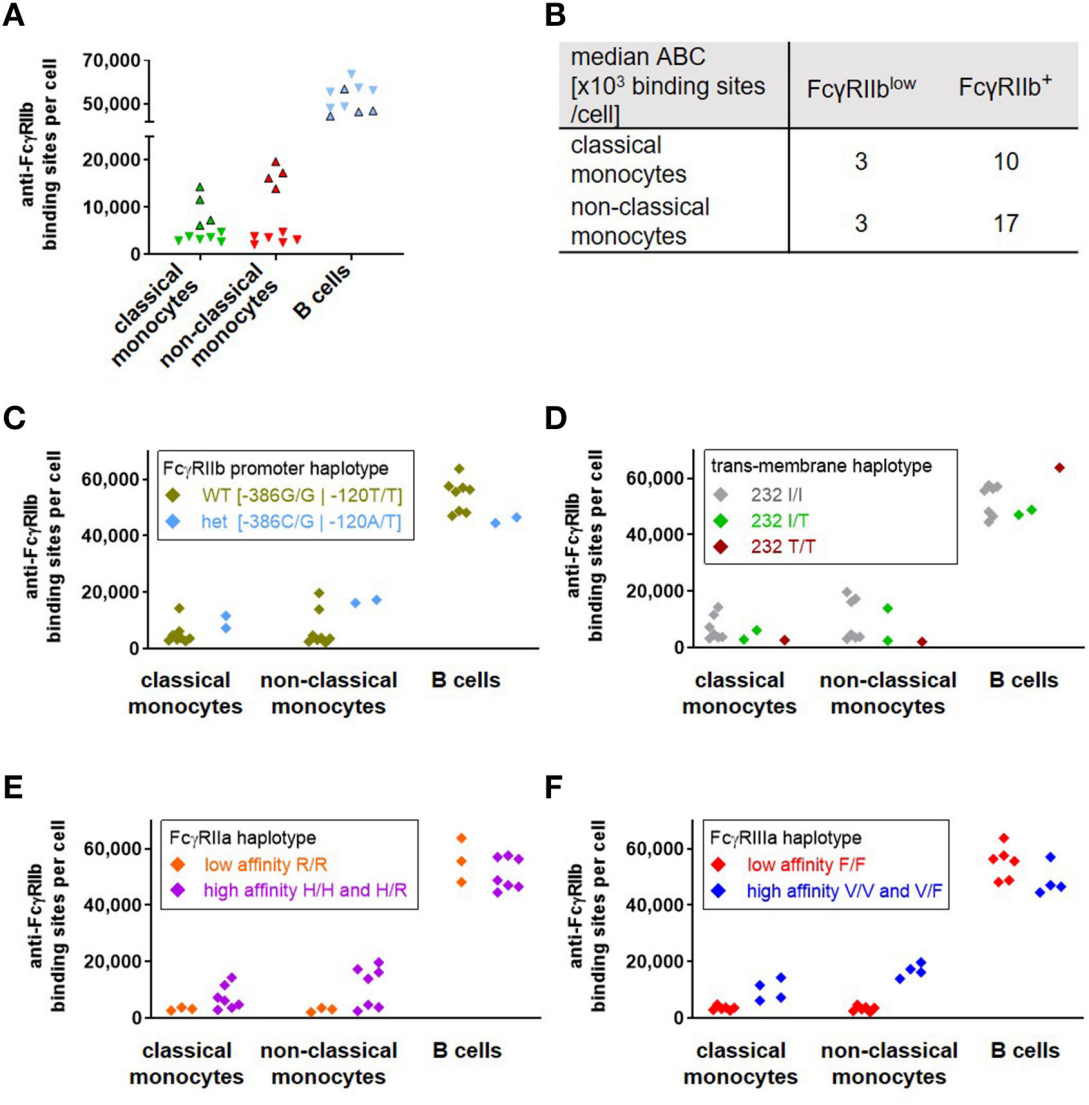
Correlation of FcγRIIb expression with Fc receptor haplotypes in human donors. As depicted in **(A)**, a group of four of ten donors (FcγRIIb^+^, bordered triangles) revealed a significantly higher expression of FcγRIIb (given as anti-FcγRIIb binding sites per cell) on monocytes than the others(FcγRIIb^low^, inverted triangles without borders). Individual values are depicted for classical monocytes (green), non-classical monocytes (red) and B cells (blue). Median anti-FcγRIIb binding sites on monocyte subsets of FcγRIIb^+^ and FcγRIIb^low^ groups are depicted in **(B)**. **(C–F)** Anti-FcγRIIb binding sites on monocytes and B cells of the ten donors are grouped according their respective haplotypes for **(C)** the coupled−386−120 promoter polymorphisms, **(D)** the isoleucine vs. threonine polymorphism at position 232 in the transmembrane region of FcγRIIb for homozygous I/I, T/T or heterozygous I/T donors, **(E)** the low affinity haplotype of FcγRIIa being homozygous with arginine at position 131 vs. the high affinity haplotype with at least one histidine allele and **(F)** the low affinity haplotype of FcγRIIIa, homozygous with phenylalanine at position 158 or the high affinity haplotype bearing at least one valine allele.

Upon quantification of FcγRIIb expression for a cohort of 10 healthy donors we found a pronounced variability in ABC values for FcγRIIb mainly on monocytes, which has also been observed by others (55, 56). Four of the donors revealed pronounced FcγRIIb expression on monocytes and were grouped as FcγRIIb^+^, whereas the others had significantly lower FcγRIIb expression (FcγRIIb^low^) (Figure 5A). Consistent with the published data, the variation was especially pronounced on CD16^+^ CD14^low^ non-classical monocytes. Whereas on classical monocytes the mean number of anti-FcγRIIb antibody binding sites was 1 × 10^4^ for FcγRIIb^+^ donors vs. 3 × 103 for the FcγRIIb^low^ group, non-classical monocytes also revealed an ABC of 3 × 103 binding sites for FcγRIIb^low^ donors but even 1.7 × 10^4^ for FcγRIIb^+^ donors (Figure 5B). Having identified the haplotypes of all donors regarding (i) the promoter polymorphism with either guanine at position −386 and thymine at position −120 or cytosine at −386 and adenine at −120 which affects transcription of the gene (57) and (ii) the transmembrane polymorphism of FcγRIIb which excludes the receptor from membrane rafts (58), we tried to correlate these haplotypes with the variances in FcγRIIb expression (Figures 5C,D). Among the 10 donors only two were heterozygous with respect to the promotor polymorphism carrying one allele of the−386C−120A haplotype whereas eight donors were homozygous “wild type” with the common−386G−120T (59). However, this promotor haplotype did not correlate with the observed dichotomy in expression on monocytes (Figure 5C). With respect to the FcγRIIb-232T variant, again no correlation with FcγRIIb expression level became apparent (58) (Figure 5D).

Since neither FcγRIIb haplotype accounted for the observed dichotomy in expression, we extended the analysis to known polymorphisms of FcγRIIa and IIIa. This includes the histidine vs. arginine polymorphism at position 131 of FcγRIIa (60) (referred to as position 133 in the original reference) as well as the valine vs. phenylalanine polymorphism at amino acid position 158 (or 176 if the leader sequence is included) of FcγRIIIa (61), which both affect IgG binding. We compared the respective high-affinity and low-affinity variants with the FcγRIIb expression (Figures 5E,F). The high affinity haplotypes for FcγRIIa carry at least one allele with a histidine at position 131 of FcγRIIa, whereas presence of two alleles encoding an arginine at this position represent the FcγRIIa low affinity haplotype (60). With respect to FcγRIIb expression, all three homozygous donors with 131R/R low affinity receptor (which, by chance, also carried the FcγRIIIa low affinity haplotype (see below)) revealed low expression of FcγRIIb on monocytes and heterogeneous expression on B cells. However, the high affinity haplotypes were heterogeneous for FcγRIIb expression on both monocytes and B cells (Figure 5E) (61). Whereas all donors with 158F/F low affinity receptors had rather low FcγRIIb expression, all those with pronounced expression of FcγRIIb carried the FcγRIIIa high affinity haplotypes (Figure 5F). For B cells we could not identify any single haplotype of those analyzed here or combinations thereof (not shown) that would correlate with the somewhat divergent expression of FcγRIIb on these cells (Figures 5C–F). Results on FcγRIIb expression from a second experiment performed for parallel quantification of activating and inhibitory receptors from six individuals (selected for containing two donors with FcγRIIIa 158 F/F, 158 V/F, and 158 V/V haplotype, respectively) were included in this data set. With the exception of a single sample with the homozygous FcγRIIIa high affinity haplotype but low FcγRIIb expression on monocytes—both datasets revealed corresponding results (Supplementary Figure 8). The individual data on age—which revealed no correlation with FcγRIIb^low^ and FcγRIIb^+^ phenotype—and FcγRIIa/FcγRIIIa haplotype from both experiments are depicted in Supplementary Figures 9A,B, respectively.

### FcγR Expression on Basophils and Platelets

**Figure 6:**
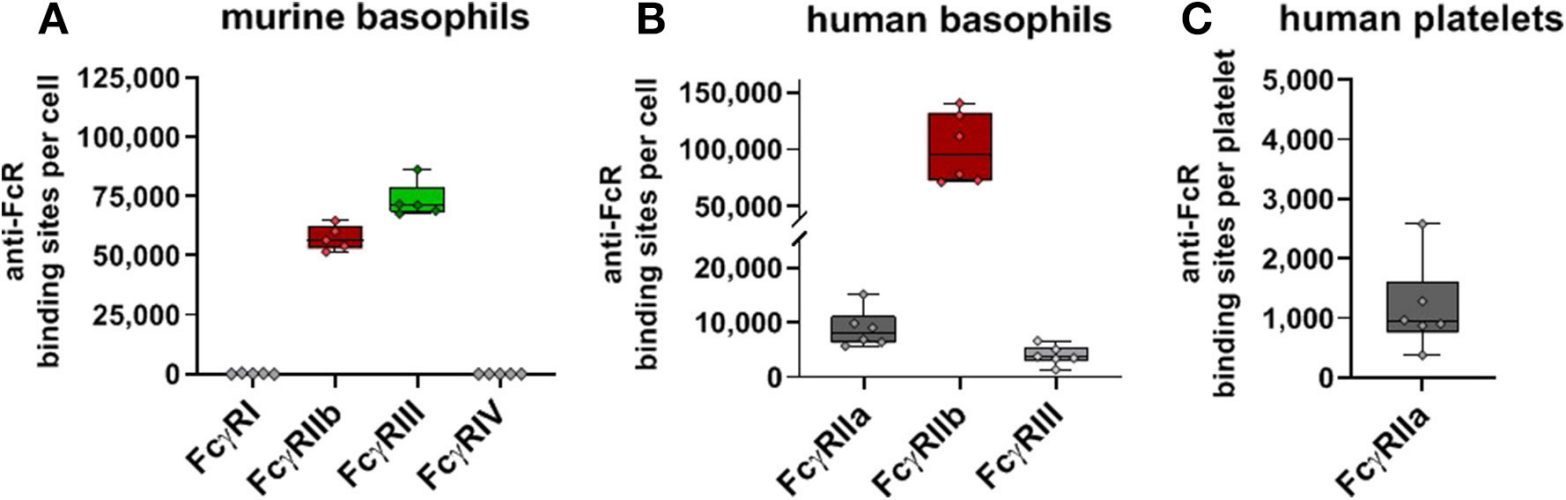
Fc gamma receptors on basophils and platelets. Depicted are box plots showing anti-FcγR binding sites for receptors expressed on **(A)** murine or **(B)** human basophils, and **(C)** human platelets from five C57BL/6 mice or six human donors, respectively.

In addition to the major leukocyte populations in the peripheral blood we also quantified Fc receptor expression on murine and human basophils as well as human platelets (Figure 6). In full accordance with previous results (62) both murine and human basophils co-express activating and inhibitory Fcγ receptors. We verified that both human and murine basophils lack FcγRI expression (not shown). With 5.6 × 10^4^ anti-FcγRIIb binding sites per cell, expression of the inhibitory receptor by murine basophils even exceeds that of B cells. This is contrasted by an even higher expression of the activating FcγRIII (7.1 × 10^4^ binding sites per cell) whereas no FcγRIV expression was detected. Also human basophils revealed high FcγRIIb expression (9.5 × 10^4^ binding sites). In contrast to murine basophils, expression of activating Fcγ receptors on human basophils is much smaller. As shown in Figure 6B, using the anti-FcγRII antibody IV.3 with pre-blocking FcγRIIb verified that the weak binding of IV.3 to basophils is not caused by low-affinity binding to FcγRIIb but indeed reflects moderate expression of FcγRIIa (8 × 103 binding sites per cell). With ~4 × 103 anti-FcγRIII binding sites per cell, FcγRIII is expressed at very low levels. Of note, results of Meknache et al. suggest that this weakly expressed FcγRIII might in fact be GPI-anchored FcγRIIIb rather than signaling competent FcγRIIIa (54).

Finally, we studied human FcγR expression on platelets. In contrast to murine thrombocytes, human platelets are known to express Fcγ receptors, namely FcγRIIa. Our quantification of FcγRIIa on human platelets revealed low ABC values with ~1 × 103 binding sites per cell. Relatively low expression of FcγRIIa by human platelets in comparison to the FcγR expressing leukocytes—has also been reported by others (1.5–4.7 × 103 binding sites) (63–65).

### Individual FcγR Expression Repertoires

Since for a cohort of six human donors the expression of FcγRI, FcγRIIa, FcγRIIb, and FcγRIII has been quantified in parallel, the respective expression pattern of the different Fcγ receptors can be assessed for each individual donor. Thus, for peripheral blood leukocytes effectively expressing more than one signaling-competent Fcγ receptor, i.e., monocytes, eosinophils, and basophils—both the relative fraction of its receptor repertoire as well as the absolute amount is shown in Supplementary Figure 10 for each individual donor. These data show that in spite of individual differences the principal A_rec_/I_rec_ with respect to predominance of either the activating Fcγ receptors (on monocytes and eosinophils) or the inhibitory receptor (on basophils) is conserved for the respective cell populations among the various donors.

## Discussion

The main purpose of our study was to quantify mouse and human FcγR expression on immune cells. From a qualitative point of view our results regarding the expression pattern of the different activating and the inhibitory FcγR on human and murine peripheral blood leukocytes correspond well with previously published data (1, 9–11, 66). However, the main purpose of the current study was to add a quantitative aspect to these relative expression values. Apart from allowing to assign numerical values for FcR expression for various cell types, this also allows to estimate ratios of activating and inhibitory receptors on a given cell population and e.g., quantitative comparison of receptor expression on murine and human leukocytes.

### Experimental Caveats

The methodology used in this study to estimate and compare numbers of binding sites for antibodies for antigens of interest is better suited than qualitative statements on differences in fluorescence intensities. However, since the binding to the respective reference beads requires complete antibody molecules it should be noted that due to the bivalent nature of antibodies one anti-FcγR antibody may theoretically bind two receptor molecules if they are in close proximity and proper orientation to one another and, thus, numbers of antibody binding sites may under-represent the numbers of respective antigens as was observed for CD4 molecules on human leukocytes (37). However, experiments performed with the immobilized antigen indicated that bivalent binding takes place only if mobility of antigens in plasma membranes is sufficiently high. Thus, in spite of staining at low temperatures in our experiments to reduce membrane mobility, there is a theoretical possibility of systemic underrepresentation of the actual number of respective Fcγ receptor molecules by the ABC up to a factor of two at most. We, thus, strictly referred to ABC/anti-FcγR binding sites per cell in the context of this work. Antibodies could also bind to Fc receptors via their Fc portion, once the Fab binds with high affinity to its antigen. However, this could only affect the quantification (leading to an underestimation of the actual receptor numbers) if the Fc-binding receptor were the Fcγ receptor of interest in the respective measurement. In this case, the lower affinity binding via an Fc part competes with the specific high affinity binding via the Fab of the anti-FcγR detection antibody. Due to the anti-FcγR antibodies being present in saturating amounts in the experiments presented here, they will probably replace virtually all Fc domains bound to the respective receptor of interest and contribute normally to the total anti-FcγR-PE mediated fluorescence of the cell. Conversely, binding of anti-FcγR antibodies via their Fc part to other Fcγ receptors than the receptor of interest would represent unspecific binding and cause overestimation of actual receptor numbers. To avoid such unspecific Fc-mediated binding in the mouse system we used well-established blocking antibodies directed against Fc receptors that were not to be quantified. For experiments with human samples we used FcX^™^ Fc Receptor Blocking Solution which limits Fc-mediated binding to FcγRs by pre-occupying their Fc binding sites, but does not affect antigen-specific FcγR-detection. In physiologically active cells, pretreatment with FcγR binding antibodies can mediate receptor crosslinking, and, thus, induce signaling and changes in surface receptor numbers. Staining at low temperatures and in presence of sodium azide renders the cells physiologically inactive to avoid such potential influences of Fc block.

In most cases, where quantitative expression data of Fcγ receptors on distinct cell populations are available in the literature, these values are exceeded by the ABC values presented in this work. Besides the possibility that such differences may be inherent to the different methodologies being used for receptor quantification, conceivable reasons for the higher values presented here may be that (i) we use freshly isolated biological samples, and (ii) we aim at saturating conditions for anti-FcγR antibody binding to cells, which is critical for assays based on antibody-binding reference beads.

### A/I Ratios of Murine Fc Receptors

Based on the different affinities of the various IgG isotypes to the activating and inhibitory Fc receptors the concept of the so called A/I ratio has been developed as a prediction of the outcome of binding of IgG molecules of a given isotype (13). The numerical data of the present work enable a corresponding concept for the ratio of activating (A_rec_) and inhibitory receptors (I_rec_) on immune cell subsets to better comprehend the roles of the distinct cell types in antibody-mediated effector functions. For example, based on our ABC values for the activating receptors FcγRI, FcγRIII and FcγRIV and inhibitory FcγRIIb, classical monocytes have an overall A_rec_/I_rec_ ratio of ~2 with Fcγ receptor III providing a somewhat higher contribution to this A_rec_/I_rec_ ratio than FcγRI. Taking into account the different affinities of these FcγRs to the various IgG isotypes, one may speculate that IgG1 or IgG2b may act mainly via FcγRIII and IgG2a may act via FcγRI. According to their respective A_rec_/I_rec_ ratios either may be controlled by comparable amounts of FcγRIIb. According to our ABC values, non-classical murine monocytes have a total A_rec_/I_rec_ ratio of ~10 with an individual contribution of ~1 by FcγRIII and ~9 by FcγRIV. Thus, due to the very low affinity of IgG1 to FcγRIV—IgG1-mediated effects may be expected to induce signaling mainly via FcγRIII, whereas IgG2a and IgG2b responses should be dominated by FcγRIV. In models where non-classical monocytes play an important role, the lack of inhibitory receptors by using FcγRIIb knockout mice should affect mainly IgG1-mediated responses but have a much weaker effect on IgG2a and IgG2b responses, especially when taking into account (and, thus, implementing the classical A/I concept) the significantly higher affinity of IgG2a to FcγRIV compared to FcγRIIb. This may e.g., be one reason for earlier observations of isotype specific effects in mice lacking inhibitory Fcγ-receptor expression in anti-tumor responses (13, 67).

This concept of cell specific A_rec_/I_rec_ ratios may be especially useful for a better understanding of effector functions mediated by subclasses with intermediate classical A/I ratio such as murine IgG2b as discussed in Nimmerjahn and Ravetch (13), or various murine or human glyco-variants, or by immune-complexes of mixed isotypes.

### FcγR Knockout Mice

It is common practice to use knockout mice lacking one or several FcγRs to study the role of these receptors in IgG dependent immune responses. However, a compensatory upregulation of other Fcγ-receptors may complicate the interpretation of the results. In mice this may be of special relevance for activating Fc-receptors as all FcγRs and the high affinity FcεRI require the common FcRγ-chain for cell surface expression and signaling function (41, 68). Indeed, this seems to be the case for activating receptors FcγRIII and FcγRIV on cells which express both molecules (Figures 2B,C). As has been suggested earlier in analogy to increased FcγRIII expression on mast cells that lack the high affinity IgE receptor FcεRI (69) this may be due to the fact that the activating receptors compete for association with the common FcRγ-chain, which is essential for expression of the activating Fc receptor α-chains on the cell surface (51, 52). The relatively low expression of FcγRI (Figure 1), however, might be the reason why its knockout does not influence the expression of FcγRIV on non-classical monocytes or neutrophils (Figure 2B) or FcγRIII on any population (Figure 2C).

### Functional Role Human and Murine NK Cells in ADCC

Both, human and murine NK cells, express only one activating receptor (FcγRIII in mice and FcγRIIIa in humans). However, in contrast to human NK cells, murine NK cells show the weakest expression of all tested FcγRIII-expressing leukocytes. This may have pronounced functional consequences: In humans, NK cells have been suggested to be key effector cells for antibody dependent cell mediated cytotoxicity (ADCC) (70). In contrast, due to their very low number of activating FcγRIII receptor molecules, a relevant role of NK cells in ADCC is unlikely in mice. This is in line with various experimental observations: (i) crosslinking of FcγRIII poorly activates murine NK cells in absence of additional stimulation (71); (ii) anti-CD20 mediated B cell depletion in mouse models is independent of NK cells but rather depends on FcγR expressing mononuclear phagocytic cells (72); (iii) anti-CD25 rat IgG1-mediated depletion of regulatory T cells is fully dependent on FcγRIII-expressing mononuclear phagocytic cells but is efficient in mice lacking NK cells (73, 74); (iv) the human anti-CCR4 monoclonal antibody KM2760 has anti-tumor activity both in murine and human experimental systems. However, whereas NK cell-mediated ADCC was suggested as the major anti-tumor effector mechanism in humans (75), in mice not NK cells but rather myeloid cells seem to be responsible for the anti-CCR4 dependent tumor cell depletion (75).

### Variability in Human FcγRIIb Expression

Upon quantification of human FcγRIIb on leukocytes we found that one group of donors showed low ABC for FcγRIIb on monocytes whereas another group showed pronounced expression. This observation is consistent with published data (i) by Bruhns et al. showing that only a fraction of human donors revealed significant FcγRIIb expression on monocytes (55) and here especially on CD16^+^ CD14^low^ non-classical monocytes [referred to as patrolling monocytes in (55)] and (ii) by Glennie et al. where variable expression of FcγRIIb was found on non-classical CD14^lo^ monocytes with lower expression on CD14^hi^ monocytes (56). Since the dichotomy of FcγRIIb ABC values was so obvious and careful assessment of FcγRIIb in the blood of patients receiving a therapeutic mAb was suggested to be an important marker of prognostic value (56) we investigated whether this variation correlated to any FcγR-related factor. Among all four haplotypes, interestingly, only FcγRIIIa variants revealed a correlation with FcγRIIb expression on monocytes: The high-affinity FcγRIIIa was present in all individuals with pronounced FcγRIIb expression, whereas all individuals with the low affinity haplotype revealed low ABC for FcγRIIb on monocytes. More studies with larger cohorts of human donors will be necessary to verify and understand this correlation in more detail in the future. Regarding the frequency and linkage of FcγRIIa and FcγRIIIa our data from the first random cohort with 10 donors, corresponds to published data on Caucasian populations. In accordance e.g., with van der Pol et al. (76) the two allelic variants of FcγRIIa are equally represented (ten 131R alleles and ten 131H alleles) among the 20 FcγRIIa alleles present in this cohort of 10 donors, whereas the 158F variant of FcγRIIIa is more common than the 158V variant (14 158F alleles and six 158V alleles). In addition, a linkage disequilibrium between FcγRIIa and FcγRIIIa became apparent: For nine of the 10 donors the allelic combinations of FcγRIIa and FcγRIIIa can be unequivocally determined since they are homozygous for at least one allele. Among these 18 allelic combinations the FcγRIIa^131H^ FcγRIIIa^158V^ (131H/158V) combination is present four times, the 131H/158F combination five times. 131R/158V and 131R/158F are present 1 and 8 times, respectively. This is in accordance with the enhanced co-occurrence of FcγRIIa^131R^ with FcγRIIIa^158F^ which has been described e.g., by Niederer et al. where a moderate/strong linkage disequilibrium between FcγRIIa and FcγRIIIa was found in a UK and Swedish Caucasian as well as a Kenyan cohort (77).

With respect to counterbalancing activating signals, it would be reasonable that cells with higher affinity activating Fcγ-receptor alleles would require a higher level of FcγRIIb expression to counter regulate this lower threshold for cell activation. Inversely, cells with low affinity activating receptor haplotypes require less pronounced counter regulation. This is in line with the low FcγRIIb expression on monocytes, consistently (but not exclusively) detectable in individuals with low affinity FcγRIIa/FcγRIIIa haplotypes. Following this line of argument, the modulation of inhibitory receptor expression by activating receptor haplotypes may well be cell type dependent, since this is relevant mainly for cell types that co-express activating and inhibitory receptors, such as monocytes. Accordingly, we found no obvious association of FcγRIIb expression with the activating receptor haplotypes. For the FcγRIIa^131H/R^ haplotypes it was recently shown also by others that the corresponding SNP (SNP rs1801274) does not affect FcγRIIb expression on B cells (78).

How genetic regulation of FcγRIIb expression by activating receptor haplotypes might be achieved on a cellular and molecular level is not yet clear. However, one might envision that the association of FcγRIIb expression and the activating Fcγ-receptor affinity haplotype could be via a promoter/enhancer SNP that modulates expression of FcγRIIb in linkage disequilibrium with coding SNPs of activating Fc receptors as has been suggested by Roederer et al. (78).

### FcγRIIIb Expression on Human Neutrophils

According to our results, FcγRIIIb seems to be expressed at exceedingly high levels by human neutrophils. This high amount of FcγRIIIb may be important to fulfill its biological function in immune-complex induced neutrophil activation. It has been suggested to be critical for tethering immune complexes to neutrophils (79). It may be a prerequisite for the proposed role of neutrophils to clear ICs under homeostatic conditions. This concept is supported by studies demonstrating low expression of FcγRIIIb to be associated with increased susceptibility to lupus nephritis and glomerulonephritis [reviewed in (80)]. Regarding neutrophil activation, a number of groups analyzed the function of the two receptors—FcγRIIa and FcγRIIIb expressed by neutrophils under steady state conditions [references in (81)]. Their tenor is that immune-complex induced activation of neutrophils requires both receptors. Since blocking of FcγRIIIb but not of FcγRIIa strongly affects immune-complex binding, FcγRIIIb is regarded as a kind of collector for immune complexes. However, the fact that FcγRIIIb is expressed in large excess compared to FcγRIIa (>10 fold according to our ABC values) raises the question by which stoichiometry and which receptor arrangement the co-engagement of both receptors with immune-complexes may take place. It should be noted that it has been reported very recently that neutrophils also carry low levels of FcγRIIIa, with this receptor being masked by the high levels of FcγRIIIb expression (82). Thus, a minor part of the anti-FcγRIII ABC values presented here may be derived from signaling-competent FcγRIIIa.

## Conclusions

In summary, we present a comprehensive and comparative numerical quantification of Fcγ receptors on the main immune cell subsets in humans and the most common laboratory mouse strain C57BL/6J. These numerical data may enable improved stoichiometric considerations on A/I ratios regarding activating and inhibitory receptors as well as improved modeling of antibody-mediated immunological processes. In addition, we emphasize that conclusions drawn from knockout animal models have to be carefully evaluated in order to avoid potential misinterpretations due to compensatory modulation of other receptors.

## Data Availability Statement

The datasets generated for this study can be found in the Figshare repository: doi: 10.6084/m9.figshare.11604201.

## Ethics Statement

The studies involving human participants were reviewed and approved by Ethics Commission of the University of Erlangen-Nürnberg. The patients/participants provided their written informed consent to participate in this study. The animal study was reviewed and approved by Government of Lower Franconia.

## Author Contributions

MB and CK performed experiments with equal contribution and analyzed data. MB and FN supervised experiments, interpreted data and wrote the manuscript.

### Conflict of Interest

The authors declare that the research was conducted in the absence of any commercial or financial relationships that could be construed as a potential conflict of interest.

## References

[1] Nimmerjahn F Ravetch JV. Fcgamma receptors: old friends and new family members. Immunity. (2006) 24:19–28. 10.1016/j.immuni.2005.11.010 16413920

[2] Hogarth PM Pietersz GA. Fc receptor-targeted therapies for the treatment of inflammation, cancer and beyond. Nat Rev Drug Discov. (2012) 11:311–31. 10.1038/nrd2909 22460124

[3] Hargreaves CE Rose-Zerilli MJJ Machado LR Iriyama C Hollox EJ Cragg MS. Fcγ receptors: genetic variation, function, and disease. Immunol Rev. (2015) 268:6–24. 10.1111/imr.12341 26497510

[4] Lanier LL Cwirla S Yu G Testi R Phillips JH. Membrane anchoring of a human IgG Fc receptor (CD16) determined by a single amino acid. Science. (1989) 246:1611–3. 10.1126/science.2531919 2531919

[5] Pincetic A Bournazos S DiLillo DJ Maamary J Wang TT Dahan R. Type I and type II Fc receptors regulate innate and adaptive immunity. Nat Immunol. (2014) 15:707–16. 10.1038/ni.2939 25045879 PMC7430760

[6] Anania JC Chenoweth AM Wines BD MarkHogarth P. The human FcγRII (CD32) family of leukocyte FCR in health and disease. Front Immunol. (2019) 10:464. 10.3389/fimmu.2019.00464 30941127 PMC6433993

[7] Daëron M. Fc receptors as adaptive immunoreceptors. Curr Top Microbiol Immunol. 382:131–64. 10.1007/978-3-319-07911-0_7 25116099 PMC7120570

[8] Bruhns P Iannascoli B England P Mancardi DA Fernandez N Jorieux S. Specificity and affinity of human Fcγ receptors and their polymorphic variants for human IgG subclasses. Blood. (2009) 113:3716–25. 10.1182/blood-2008-09-179754 19018092

[9] Bruhns P Jönsson F. Mouse and human FcR effector functions. Immunol Rev. (2015) 268:25–51. 10.1111/imr.12350 26497511

[10] Rosales C. Fcγ receptor heterogeneity in leukocyte functional responses. Front Immunol. (2017) 8:280. 10.3389/fimmu.2017.00280 28373871 PMC5357773

[11] Dahal LN Roghanian A Beers SA Cragg MS. FcγR requirements leading to successful immunotherapy. Immunol Rev. (2015) 268:104–22. 10.1111/imr.12342 26497516

[12] Nimmerjahn F Ravetch JV. FcyRs in health and disease. Curr Top Microbiol Immunol. (2011) 350:105–25. 10.1007/82_2010_86 20680807

[13] Nimmerjahn F Ravetch JV. Immunology: divergent immunoglobulin G subclass activity through selective Fc receptor binding. Science. (2005) 310:1510–2. 10.1126/science.1118948 16322460

[14] Beers SA Glennie MJ White AL. Influence of immunoglobulin isotype on therapeutic antibody function. Blood. (2016) 127:1097–101. 10.1182/blood-2015-09-625343 26764357 PMC4797141

[15] Teige I Mårtensson L Frendéus BL. Targeting the antibody checkpoints to enhance cancer immunotherapy-focus on FcγRIIb. Front Immunol. (2019) 10:481. 10.3389/fimmu.2019.00481 30930905 PMC6423481

[16] Robinett RA Guan N Lux A Biburger M Nimmerjahn F Meyer AS. Dissecting FcγR regulation through a multivalent binding model. Cell Syst. (2018) 7:41–8.e5. 10.1016/j.cels.2018.05.018 29960887 PMC6062446

[17] Lux A Yu X Scanlan CN Nimmerjahn F. Impact of immune complex size and glycosylation on IgG binding to human FcγRs. J Immunol. (2013) 190:4315–23. 10.4049/jimmunol.1200501 23509345

[18] Antal-Szalmas P Strijp JA Weersink AJL Verhoef J Van Kessel KPM Van Strijp JAG. Quantitation of surface CD14 on human monocytes and neutrophils. J Leukoc Biol. (1997) 61:721–8. 10.1002/jlb.61.6.721 9201263

[19] Maeda M van Schie RC Yüksel B Greenough A Fanger MW Guyre PM. Differential expression of Fc receptors for IgG by monocytes and granulocytes from neonates and adults. Clin Exp Immunol. (1996) 103:343–7. 10.1046/j.1365-2249.1996.d01-615.x 8565322 PMC2200344

[20] Matsushita S Inagaki Y Nakatsuji Y Endo M Ito S. To use CD64 expression level on neutrophils as an infection marker in children 10 years old or younger cut-off values higher than that in adults must be established. Blood. (2011) 118:4930. 10.1182/blood.V118.21.4930.493021881048

[21] Katoh N Nishimura K Kawabata C Hotta Y Nakamura S Matsushita T. Normal sequential changes in neutrophil CD64 expression after total joint arthroplasty. J Orthop Sci. (2013) 18:949–54. 10.1007/s00776-013-0451-9 23943224 PMC3838574

[22] Yeager MP Colacchio TA Yu CT Hildebrandt L Howell AL Weiss J. Morphine inhibits spontaneous and cytokine-enhanced natural killer cell cytotoxicity in volunteers. Anesthesiology. (1995) 83:500–8. 10.1097/00000542-199509000-00008 7661350

[23] Guyre PM Campbell AS Kniffin WD Fanger MW. Monocytes and polymorphonuclear neutrophils of patients with streptococcal pharyngitis express increased numbers of type I IgG Fc receptors. J Clin Invest. (1990) 86:1892–6. 10.1172/JCI114921 2147695 PMC329823

[24] Petroni KC Shen L Guyre PM. Modulation of human polymorphonuclear leukocyte IgG Fc receptors and Fc receptor-mediated functions by IFN-gamma and glucocorticoids. J Immunol. (1988) 140:3467–72. 2966197

[25] Goulding NJ Knight SM Godolphin JL Guyre PM. Increase in neutrophil Fc gamma receptor I expression following interferon gamma treatment in rheumatoid arthritis. Ann Rheum Dis. (1992) 51:465–8. 10.1136/ard.51.4.465 1534001 PMC1004692

[26] Fleit HB Wright SD Unkeless JC. Human neutrophil Fc gamma receptor distribution and structure. Proc Natl Acad Sci U.S.A. (1982) 79:3275–9. 10.1073/pnas.79.10.3275 6808506 PMC346398

[27] Perussia B Dayton ET Lazarus R Fanning V Trinchieri G. Immune interferon induces the receptor for monomeric IgG1 on human monocytic and myeloid cells. J Exp Med. (1983) 158:1092–113. 10.1084/jem.158.4.1092 6225822 PMC2187379

[28] Matsui T Ohsumi K Ozawa N Shimada K Sumitomo S Shimane K. CD64 on neutrophils is a sensitive and specific marker for detection of infection in patients with rheumatoid arthritis. J Rheumatol. (2006) 33:2416–24. 17080517

[29] Cardelli P Ferraironi M Amodeo R Tabacco F De Blasi RA Nicoletti M. Evaluation of neutrophil CD64 expression and procalcitonin as useful markers in early diagnosis of sepsis. Int J Immunopathol Pharmacol. (2008) 21:43–9. 10.1177/039463200802100106 18336730

[30] Hardin JA Downs JT. Saturable, high-avidity monocyte receptors for monomeric IgG and Fc fragments increase in SLE and lyme disease. Clin Exp Rheumatol. (1983) 1:327–32. 6241857

[31] Rossman MD Chien P Cassizzi-Cprek A Elias JA Holian A Schreiber AD. The binding of monomeric IgG to human blood monocytes and alveolar macrophages. Am Rev Respir Dis. (1986) 133:292–7. 3080932 10.1164/arrd.1986.133.2.292

[32] Christensen J Leslie RG. Quantitative measurement of Fc receptor activity on human peripheral blood monocytes and the monocyte-like cell line, U937, by laser flow cytometry. J Immunol Methods. (1990) 132:211–9. 10.1016/0022-1759(90)90032-Q 2145369

[33] Fries LF Brickman CM Frank MM. Monocyte receptors for the Fc portion of IgG increase in number in autoimmune hemolytic anemia and other hemolytic states and are decreased by glucocorticoid therapy. J Immunol. (1983) 131:1240–5. 6224854

[34] Huizinga TW Kerst M Nuyens JH Vlug A von dem Borne AE Roos D. Binding characteristics of dimeric IgG subclass complexes to human neutrophils. J Immunol. (1989) 142:2359–64. 2784461

[35] Bikoue A George F Poncelet P Mutin M Janossy G Sampol J. Quantitative analysis of leukocyte membrane antigen expression: normal adult values. Cytometry. (1996) 26:137–47. 10.1002/(SICI)1097-0320(19960615)26:2 <137::AID-CYTO7>3.0.CO;2-D 8817090

[36] Hardy RR. Purification and coupling of fluorescent proteins for use in flow cytometry. In: Weir DM Herzenberg LA Blackwell C Herzenberg LA editors. Handbook of Experimental Immunology, 4th ed. Boston, MA: Blackwell Scientific Publications (1986).

[37] Davis KA Abrams B Iyer SB Hoffman RA Bishop JE. Determination of CD4 antigen density on cells: Role of antibody valency, avidity, clones, and conjugation. Cytometry. (1998) 33:197–205. 10.1002/(sici)1097-0320(19981001)33:2<197::aid-cyto14>3.0.co;2-p 9773880

[38] Gordan S Biburger M Nimmerjahn F. bIgG time for large eaters: monocytes and macrophages as effector and target cells of antibody-mediated immune activation and repression. Immunol Rev. (2015) 268:52–65. 10.1111/imr.12347 26497512

[39] Barnes N Gavin AL Tan PS Mottram P Koentgen F Hogarth PM. FcgammaRI-deficient mice show multiple alterations to inflammatory and immune responses. Immunity. (2002) 16:379–89. 10.1016/S1074-7613(02)00287-X 11911823

[40] Li F Ravetch JV. Apoptotic and antitumor activity of death receptor antibodies require inhibitory Fcγ receptor engagement. Proc Natl Acad Sci U.S.A. (2012) 109:10966–71. 10.1073/pnas.1208698109 22723355 PMC3390832

[41] Nimmerjahn F Bruhns P Horiuchi K Ravetch JV. FcgammaRIV: a novel FcR with distinct IgG subclass specificity. Immunity. (2005) 23:41–51. 10.1016/j.immuni.2005.05.010 16039578

[42] Unkeless JC. Characterization of a monoclonal antibody directed against mouse macrophage and lymphocyte Fc receptors. J Exp Med. (1979) 150:580–96. 10.1084/jem.150.3.580 90108 PMC2185638

[43] Biburger M Trenkwald I Nimmerjahn F. Three blocks are not enough-Blocking of the murine IgG receptor FcγRIV is crucial for proper characterization of cells by FACS analysis. Eur J Immunol. (2015) 45:2694–7. 10.1002/eji.201545463 26138319

[44] Walker MR Lund J Thompson KM Jefferis R. Aglycosylation of human IgG1 and IgG3 monoclonal antibodies can eliminate recognition by human cells expressing FcγRI and/or FcγRII receptors. Biochem J. (1989) 259:347–353. 10.1042/bj2590347 2524188 PMC1138517

[45] Shields RL Namenuk AK Hong K Meng YG Rae J Briggs J. High resolution mapping of the binding site on human IgG1 for FcγRI, FcγRII, FcγRIII, and FcRn and design of IgG1 variants with improved binding to the FcγR. J Biol Chem. (2001) 276:6591–604. 10.1074/jbc.M009483200 11096108

[46] Rankin CT Veri M-C Gorlatov S Tuaillon N Burke S Huang L. CD32B, the human inhibitory Fc-gamma receptor IIB, as a target for monoclonal antibody therapy of B-cell lymphoma. Blood. (2006) 108:2384–91. 10.1182/blood-2006-05-020602 16757681

[47] Deka C Lehnert BE Lehnert NM Jones GM Sklar LA Steinkamp JA. Analysis of fluorescence lifetime and quenching of FITC-conjugated antibodies on cells by phase-sensitive flow cytometry. Cytometry. (1996) 25:271–9. 10.1002/(SICI)1097-0320(19961101)25:3<271::AID-CYTO8>3.0.CO;2-I 8914824

[48] Lehmann B Biburger M Brückner C Ipsen-escobedo A Gordan S Lehmann C. Tumor location determines tissue-specific recruitment of tumor-associated macrophages and antibody-dependent immunotherapy response. Sci Immunol. (2017) 2:eaah6413. 10.1126/sciimmunol.aah6413 28783667

[49] Yona S Kim K-W Wolf Y Mildner A Varol D Breker M. Fate mapping reveals origins and dynamics of monocytes and tissue macrophages under homeostasis. Immunity. (2013) 38:79–91. 10.1016/j.immuni.2012.12.001 23273845 PMC3908543

[50] Biburger M Nimmerjahn F. Low level of FcgammaRIII expression on murine natural killer cells. Immunol Lett. (2012) 143:53–9. 10.1016/j.imlet.2012.01.002 22285694

[51] Syed SN Konrad S Wiege K Nieswandt B Nimmerjahn F Schmidt RE. Both FcgammaRIV and FcgammaRIII are essential receptors mediating type II and type III autoimmune responses via FcRgamma-LAT-dependent generation of C5a. Eur J Immunol. (2009) 39:3343–56. 10.1002/eji.200939884 19795417

[52] Nimmerjahn F Lux A Albert H Woigk M Lehmann C Dudziak D. Fc RIV deletion reveals its central role for IgG2a and IgG2b activity *in vivo*. Proc Natl Acad Sci U.S.A. (2010) 107:19396–401. 10.1073/pnas.1014515107 20974962 PMC2984189

[53] Beutier H Gillis CM Iannascoli B Godon O England P Sibilano R. IgG subclasses determine pathways of anaphylaxis in mice. J Allergy Clin Immunol. (2017) 139:269–80.e7. 10.1016/j.jaci.2016.03.028 27246523 PMC5081282

[54] Meknache N Jönsson F Laurent J Guinnepain M-T Daëron M. Human basophils express the glycosylphosphatidylinositol-anchored low-affinity IgG receptor FcγRIIIB (CD16B). J Immunol. (2009) 182:2542–50. 10.4049/jimmunol.0801665 19201911

[55] Gillis CM Jönsson F Mancardi DA Tu N Beutier H Van Rooijen N. Mechanisms of anaphylaxis in human low-affinity IgG receptor locus knock-in mice. J Allergy Clin Immunol. (2017) 139:1253–65.e14. 10.1016/j.jaci.2016.06.058 27568081

[56] Tutt AL James S Laversin SA Tipton TR Ashton-Key M French RR. Development and characterization of monoclonal antibodies specific for mouse and human Fcγ receptors. J Immunol. (2015) 195:5503–16. 10.4049/jimmunol.1402988 26512139

[57] Su K Wu J Edberg JC Li X Ferguson P Cooper GS. A promoter haplotype of the immunoreceptor tyrosine-based inhibitory motif-bearing Fc RIIb alters receptor expression and associates with autoimmunity. I. Regulatory FCGR2B polymorphisms and their association with systemic lupus erythematosus. J Immunol. (2004) 172:7186–91. 10.4049/jimmunol.172.11.7186 15153543

[58] Floto RA Clatworthy MR Heilbronn KR Rosner DR MacAry PA Rankin A. Loss of function of a lupus-associated FcgammaRIIb polymorphism through exclusion from lipid rafts. Nat Med. (2005) 11:1056–8. 10.1038/nm1288 16170323

[59] Su K Li X Edberg JC Wu J Ferguson P Kimberly RP. A promoter haplotype of the immunoreceptor tyrosine-based inhibitory motif-bearing FcgammaRIIb alters receptor expression and associates with autoimmunity. II. Differential binding of GATA4 and Yin-Yang1 transcription factors and correlated receptor expression and function. J Immunol. (2004) 172:7192–9. 10.4049/jimmunol.172.11.7192 15153544

[60] Warmerdam PA van de Winkel JG Vlug A Westerdaal NA Capel PJ. A single amino acid in the second Ig-like domain of the human Fc gamma receptor II is critical for human IgG2 binding. J Immunol. (1991) 147:1338–43. 1831223

[61] Wu J Edberg JC Redecha PB Bansal V Guyre PM Coleman K. A novel polymorphism of FcgammaRIIIa (CD16) alters receptor function and predisposes to autoimmune disease. J Clin Invest. (1997) 100:1059–70. 10.1172/JCI119616 9276722 PMC508280

[62] Cassard L Jönsson F Arnaud S Daëron M. Fcγ receptors inhibit mouse and human basophil activation. J Immunol. (2012) 189:2995–3006. 10.4049/jimmunol.1200968 22908332

[63] Karas SP Rosse WF Kurlander RJ. Characterization of the IgG-Fc receptor on human platelets. Blood. (1982) 60:1277–82. 10.1182/blood.V60.6.1277.1277 6215962

[64] King M McDermott P Schreiber AD. Characterization of the Fc gamma receptor on human platelets. Cell Immunol. (1990) 128:462–79. 10.1016/0008-8749(90)90041-O 2141549

[65] Tomiyama Y Kunicki TJ Zipf TF Ford SB Aster RH. Response of human platelets to activating monoclonal antibodies: importance of Fc-gammaRII (CD32) phenotype and level of expression. Blood. (1992) 80:2261–8. 10.1182/blood.V80.9.2261.2261 1421396

[66] Bruhns P. Properties of mouse and human IgG receptors and their contribution to disease models. Blood. (2012) 119:5640–9. 10.1182/blood-2012-01-380121 22535666

[67] Clynes RA Towers TL Presta LG Ravetch JV. Inhibitory Fc receptors modulate *in vivo* cytotoxicity against tumor targets. Nat Med. (2000) 6:443–6. 10.1038/74704 10742152

[68] Takai T Li M Sylvestre D Clynes R Ravetch JV. FcR gamma chain deletion results in pleiotrophic effector cell defects. Cell. (1994) 76:519–29. 10.1016/0092-8674(94)90115-5 8313472

[69] Dombrowicz D Flamand V Miyajima I Ravetch JV Galli SJ Kinet JP. Absence of Fc epsilonRI alpha chain results in upregulation of Fc gammaRIII-dependent mast cell degranulation and anaphylaxis. Evidence of competition between Fc epsilonRI and Fc gammaRIII for limiting amounts of FcR beta and gamma chains. J Clin Invest. (1997) 99:915–25. 10.1172/JCI119256 9062349 PMC507899

[70] Bryceson YT March ME Ljunggren H-G Long EO. Synergy among receptors on resting NK cells for the activation of natural cytotoxicity and cytokine secretion. Blood. (2006) 107:159–66. 10.1182/blood-2005-04-1351 16150947 PMC1895346

[71] Cheung JC Koh CY Gordon BE Wilder JA Yuan D. The mechanism of activation of NK-cell IFN-gamma production by ligation of CD28. Mol Immunol. (1999) 36:361–72. 10.1016/S0161-5890(99)00051-6 10444000

[72] Uchida J Hamaguchi Y Oliver JA Ravetch JV Poe JC Haas KM. The innate mononuclear phagocyte network depletes B lymphocytes through Fc receptor-dependent mechanisms during anti-CD20 antibody immunotherapy. J Exp Med. (2004) 199:1659–69. 10.1084/jem.20040119 15210744 PMC2212805

[73] Setiady YY Coccia JA Park PU. *In vivo* depletion of CD4 + FOXP3 + Treg cells by the PC61 anti-CD25 monoclonal antibody is mediated by FcγRIII + phagocytes. Eur J Immunol. (2010) 40:780–6. 10.1002/eji.200939613 20039297

[74] Zhang M Zhang Z Garmestani K Goldman CK Ravetch JV Brechbiel MW. Activating Fc receptors are required for antitumor efficacy of the antibodies directed toward CD25 in a murine model of adult T-cell leukemia. Cancer Res. (2004) 64:5825–9. 10.1158/0008-5472.CAN-04-1088 15313926

[75] Ishida T Ishii T Inagaki A Yano H Kusumoto S Ri M. The CCR4 as a novel-specific molecular target for immunotherapy in Hodgkin lymphoma. Leukemia. (2006) 20:2162–8. 10.1038/sj.leu.2404415 17039235

[76] van der Pol W-LL Jansen MD Sluiter WJ Van De Sluis B Leppers-van de Straat FGJJ Kobayashi T. Evidence for non-random distribution of Fcγ receptor genotype combinations. Immunogenetics. (2003) 55:240–6. 10.1007/s00251-003-0574-9 12830330

[77] Niederer HA Willcocks LC Rayner TF Yang W Lau YL Williams TN. Copy number, linkage disequilibrium and disease association in the FCGR locus. Hum Mol Genet. (2010) 19:3282–94. 10.1093/hmg/ddq216 20508037 PMC2908468

[78] Roederer M Quaye L Mangino M Beddall MH Mahnke Y Chattopadhyay P. The genetic architecture of the human immune system: a bioresource for autoimmunity and disease pathogenesis. Cell. (2015) 161:387–403. 10.1016/j.cell.2015.02.046 25772697 PMC4393780

[79] Coxon A Cullere X Knight S Sethi S Wakelin MW Stavrakis G. FcγRIII mediates neutrophil recruitment to immune complexes. Immunity. (2001) 14:693–704. 10.1016/S1074-7613(01)00150-9 11420040

[80] Mayadas TN Tsokos GC Tsuboi N. Mechanisms of immune complex–mediated neutrophil recruitment and tissue injury. Circulation. (2009) 120:2012–24. 10.1161/CIRCULATIONAHA.108.771170 19917895 PMC2782878

[81] Jakus Z Németh T Verbeek JS Mócsai A. Critical but overlapping role of FcgammaRIII and FcgammaRIV in activation of murine neutrophils by immobilized immune complexes. J Immunol. (2008) 180:618–29. 10.4049/jimmunol.180.1.618 18097064 PMC2647079

[82] Golay J Valgardsdottir R Musaraj G Giupponi D Spinelli O Introna M. Human neutrophils express low levels of FcγRIIIA, which plays a role in PMN activation. Blood. (2019) 133:1395–1405. 10.1182/blood-2018-07-864538 30655272 PMC6484458

