# Corrigendum: There Is (Scientific) Strength in Numbers: A Comprehensive Quantitation of Fc Gamma Receptor Numbers on Human and Murine Peripheral Blood Leukocytes

**DOI:** 10.3389/fimmu.2021.840026

**Published:** 2022-01-20

**Authors:** Christina Kerntke, Falk Nimmerjahn, Markus Biburger

**Affiliations:** ^1^ Division of Genetics, Department of Biology, Friedrich-Alexander University Erlangen-Nürnberg, Erlangen, Germany; ^2^ Medical Immunology Campus Erlangen, Friedrich-Alexander University Erlangen-Nürnberg, Erlangen, Germany

**Keywords:** Fc receptors, antibodies, human leukocytes, murine leukocytes, quantification, receptor numbers, neutrophils, monocytes

In the original article, an error occurred in **Figure 1E**. The median ABC [x10³ binding sites/cell] for anti-FcγRI on neutrophils was shown as “**71**”. The correct number is “**<1**”, in agreement with the data presented in **Figure 1A** and deposited in the Figshare repository as well as the description in the text that among peripheral blood leukocytes of mice expression of FcγRI is restricted to monocytes. The corrected **Figure 1E** appears below.

**Figure 1:**
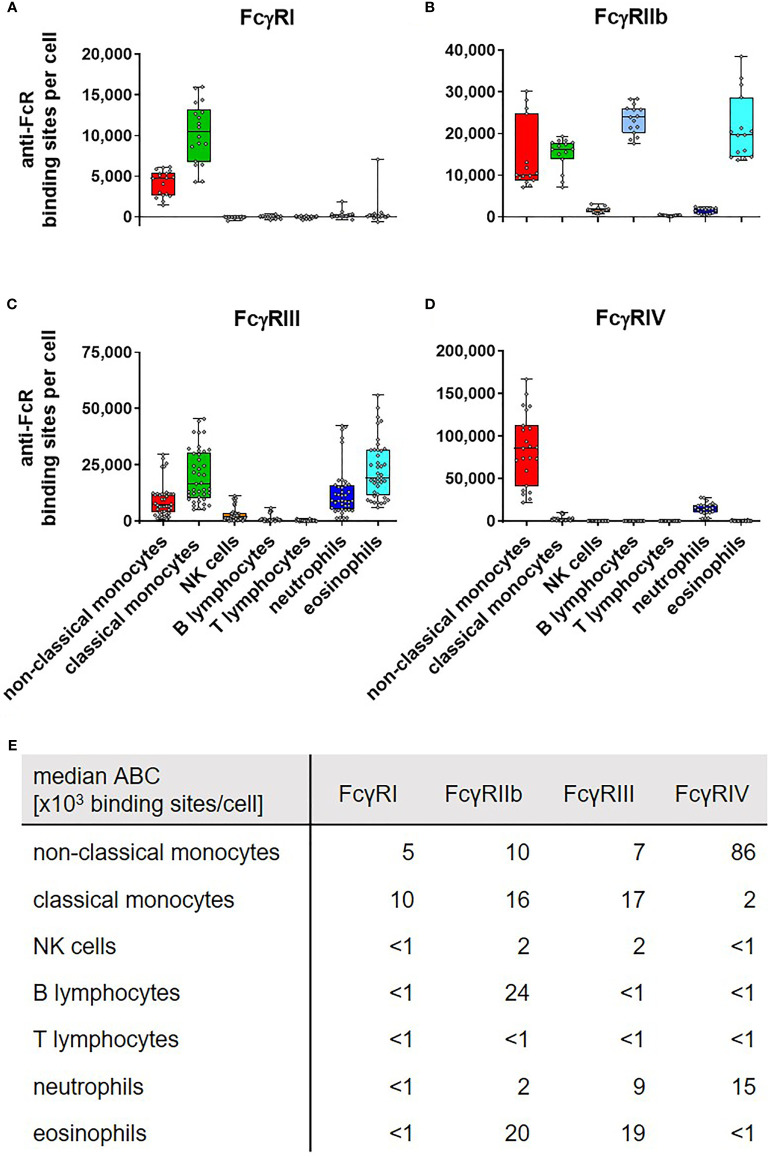
Fc gamma receptors on murine peripheral blood leukocytes. Depicted are box plots showing anti-FcR binding sites for **(A)** FcγRI, **(B)** FcγRIIb, **(C)** FcγRIII, and **(D)** FcγRIV on indicated leukocyte subsets together with **(E)** a tabular presentation of the median number of binding sites. n = 15–41 from 4 to 11 independent experiments.

The authors apologize for this error and state that this does not change the scientific conclusions of the article in any way. The original article has been updated.

## Publisher’s Note

All claims expressed in this article are solely those of the authors and do not necessarily represent those of their affiliated organizations, or those of the publisher, the editors and the reviewers. Any product that may be evaluated in this article, or claim that may be made by its manufacturer, is not guaranteed or endorsed by the publisher.

