# Supplementary material for: There Is (Scientific) Strength in Numbers: A Comprehensive Quantitation of Fc Gamma Receptor Numbers on Human and Murine Peripheral Blood Leukocytes
Source: Front Immunol. 2020 Feb 5;11:118. doi: 10.3389/fimmu.2020.00118 (PMC7013094; doi:10.3389/fimmu.2020.00118)
Supplement: Supplementary file 1 [file Presentation_1.pptx]

## Slide 1
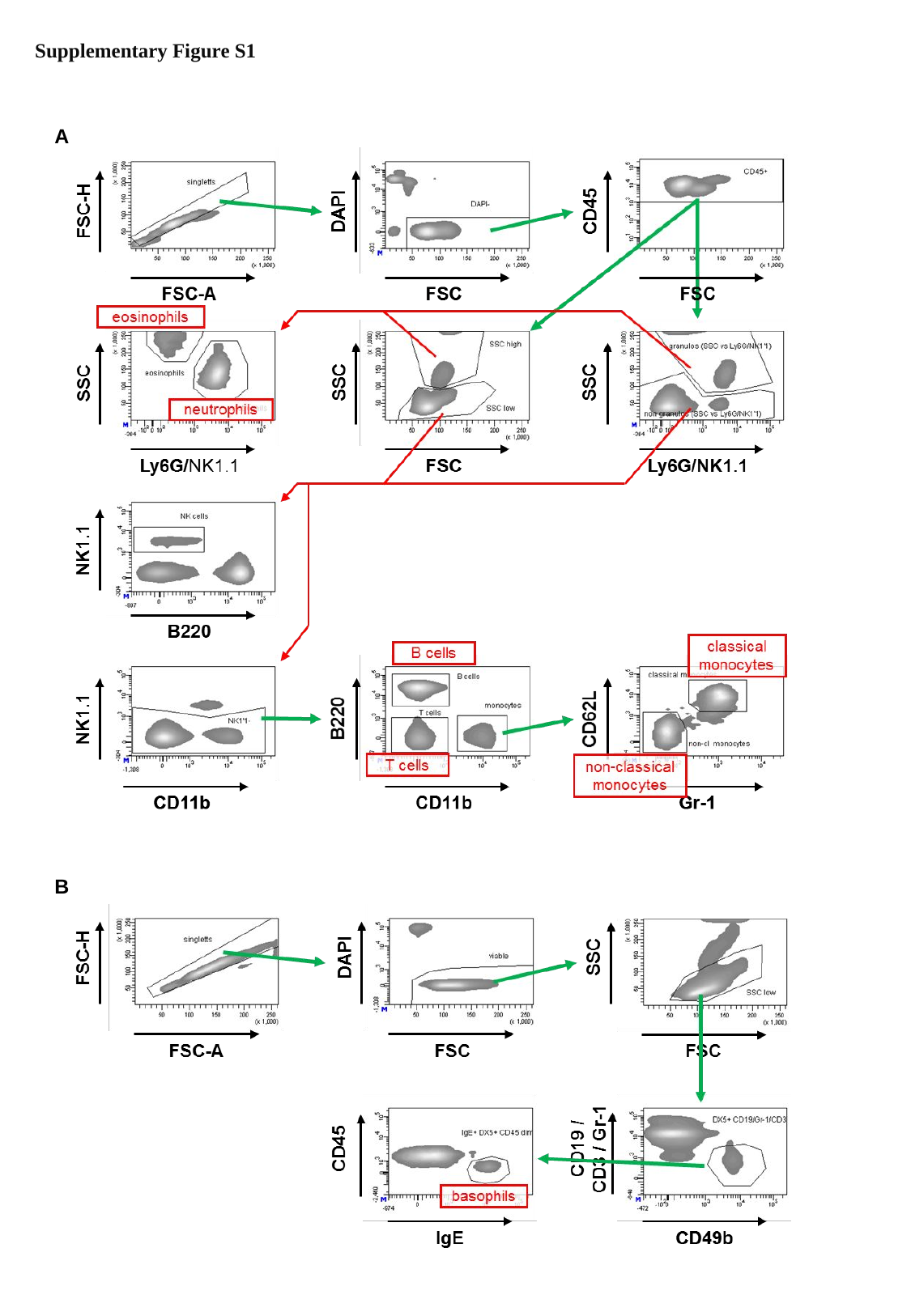

Supplementary Figure S1
A
B

## Slide 2
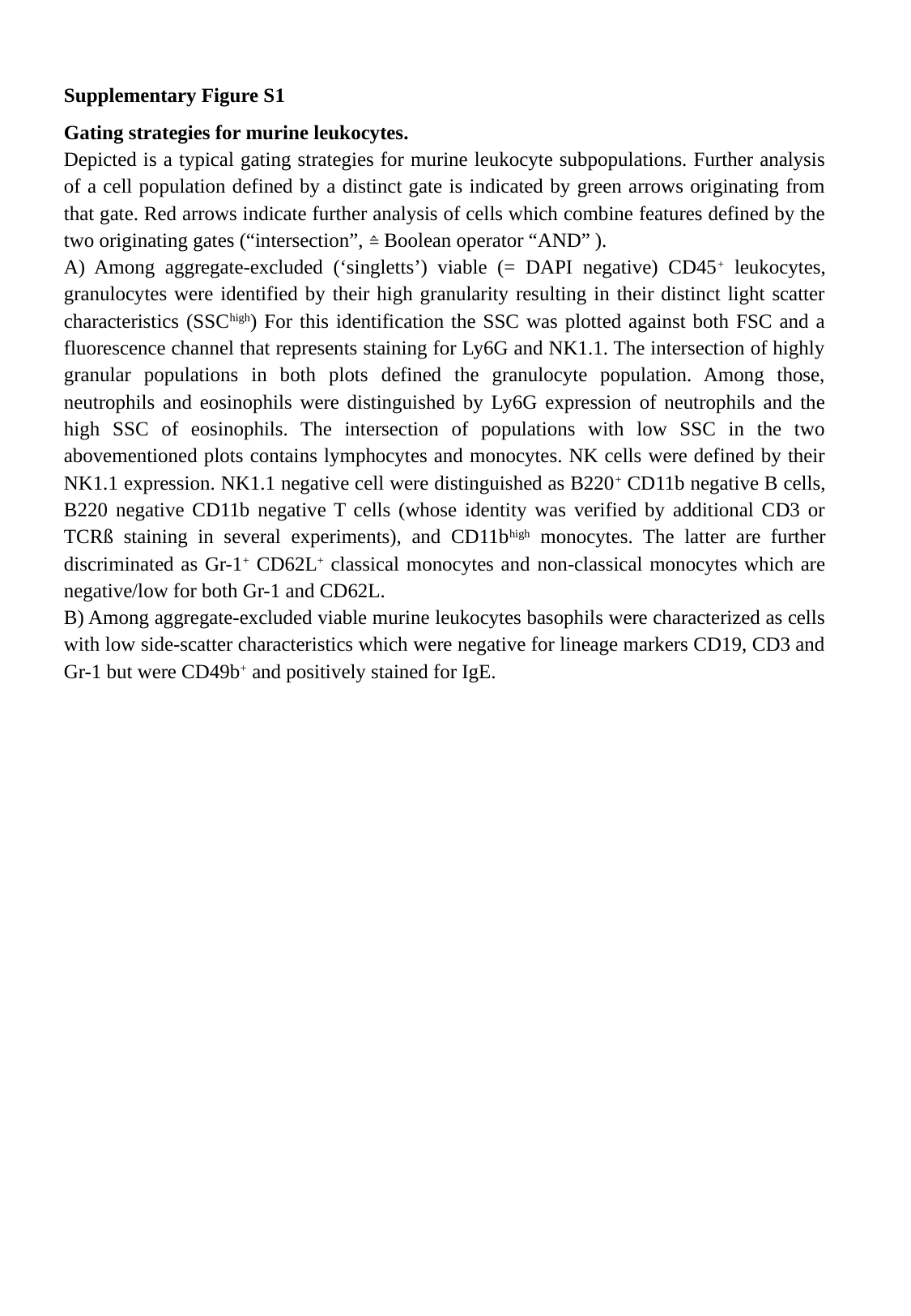

Supplementary Figure S1
Gating strategies for murine leukocytes.
Depicted is a typical gating strategies for murine leukocyte subpopulations. Further analysis of a cell population defined by a distinct gate is indicated by green arrows originating from that gate. Red arrows indicate further analysis of cells which combine features defined by the two originating gates (“intersection”, ≙ Boolean operator “AND” ).
A) Among aggregate-excluded (‘singletts’) viable (= DAPI negative) CD45+ leukocytes, granulocytes were identified by their high granularity resulting in their distinct light scatter characteristics (SSChigh) For this identification the SSC was plotted against both FSC and a fluorescence channel that represents staining for Ly6G and NK1.1. The intersection of highly granular populations in both plots defined the granulocyte population. Among those, neutrophils and eosinophils were distinguished by Ly6G expression of neutrophils and the high SSC of eosinophils. The intersection of populations with low SSC in the two abovementioned plots contains lymphocytes and monocytes. NK cells were defined by their NK1.1 expression. NK1.1 negative cell were distinguished as B220+ CD11b negative B cells, B220 negative CD11b negative T cells (whose identity was verified by additional CD3 or TCRß staining in several experiments), and CD11bhigh monocytes. The latter are further discriminated as Gr-1+ CD62L+ classical monocytes and non-classical monocytes which are negative/low for both Gr-1 and CD62L.
B) Among aggregate-excluded viable murine leukocytes basophils were characterized as cells with low side-scatter characteristics which were negative for lineage markers CD19, CD3 and Gr-1 but were CD49b+ and positively stained for IgE.

## Slide 3
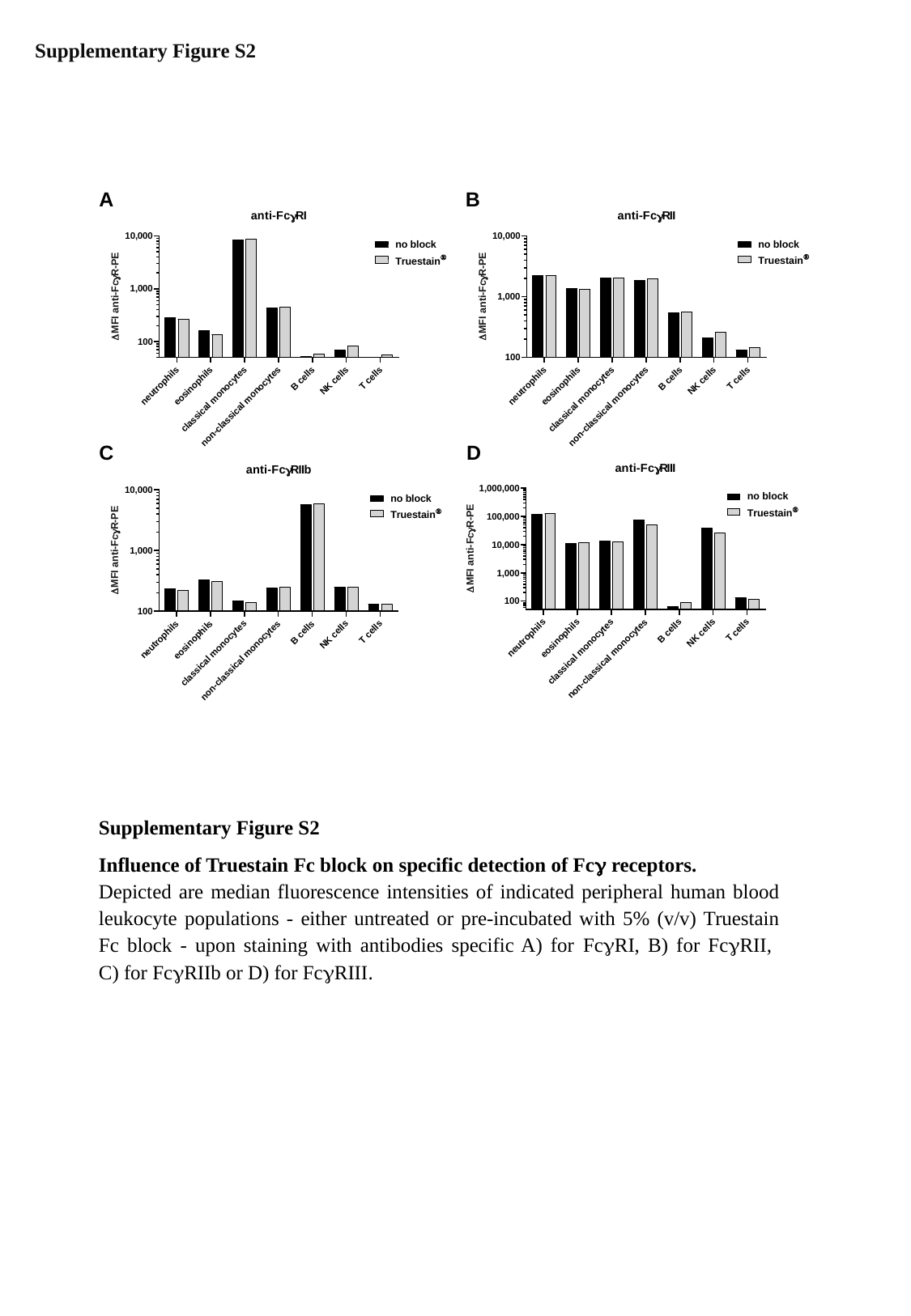

Supplementary Figure S2
B
A
C
D
Supplementary Figure S2
Influence of Truestain Fc block on specific detection of Fcγ receptors.
Depicted are median fluorescence intensities of indicated peripheral human blood leukocyte populations - either untreated or pre-incubated with 5% (v/v) Truestain Fc block - upon staining with antibodies specific A) for FcγRI, B) for FcγRII,
C) for FcγRIIb or D) for FcγRIII.

## Slide 4
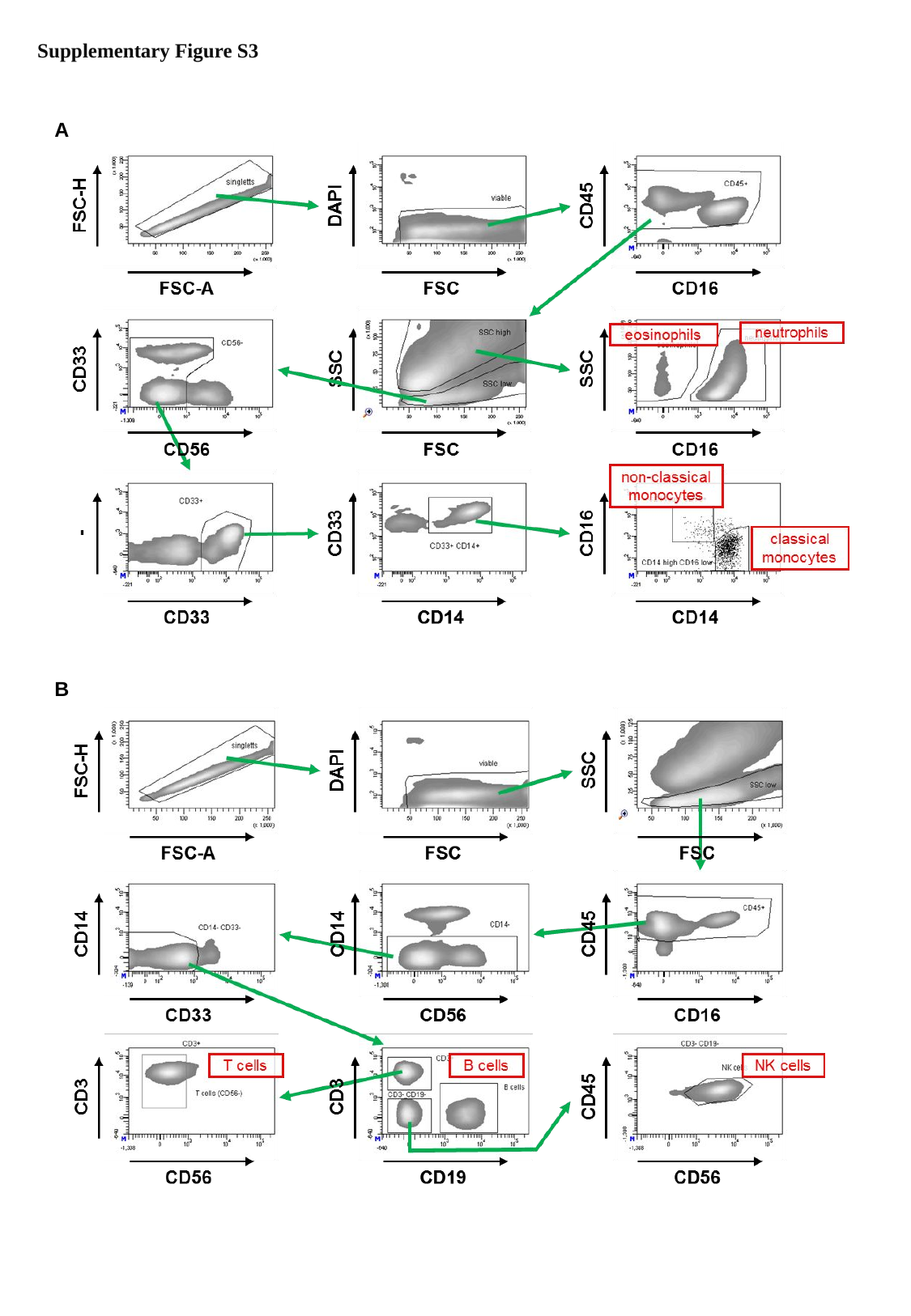

Supplementary Figure S3
A
B

## Slide 5
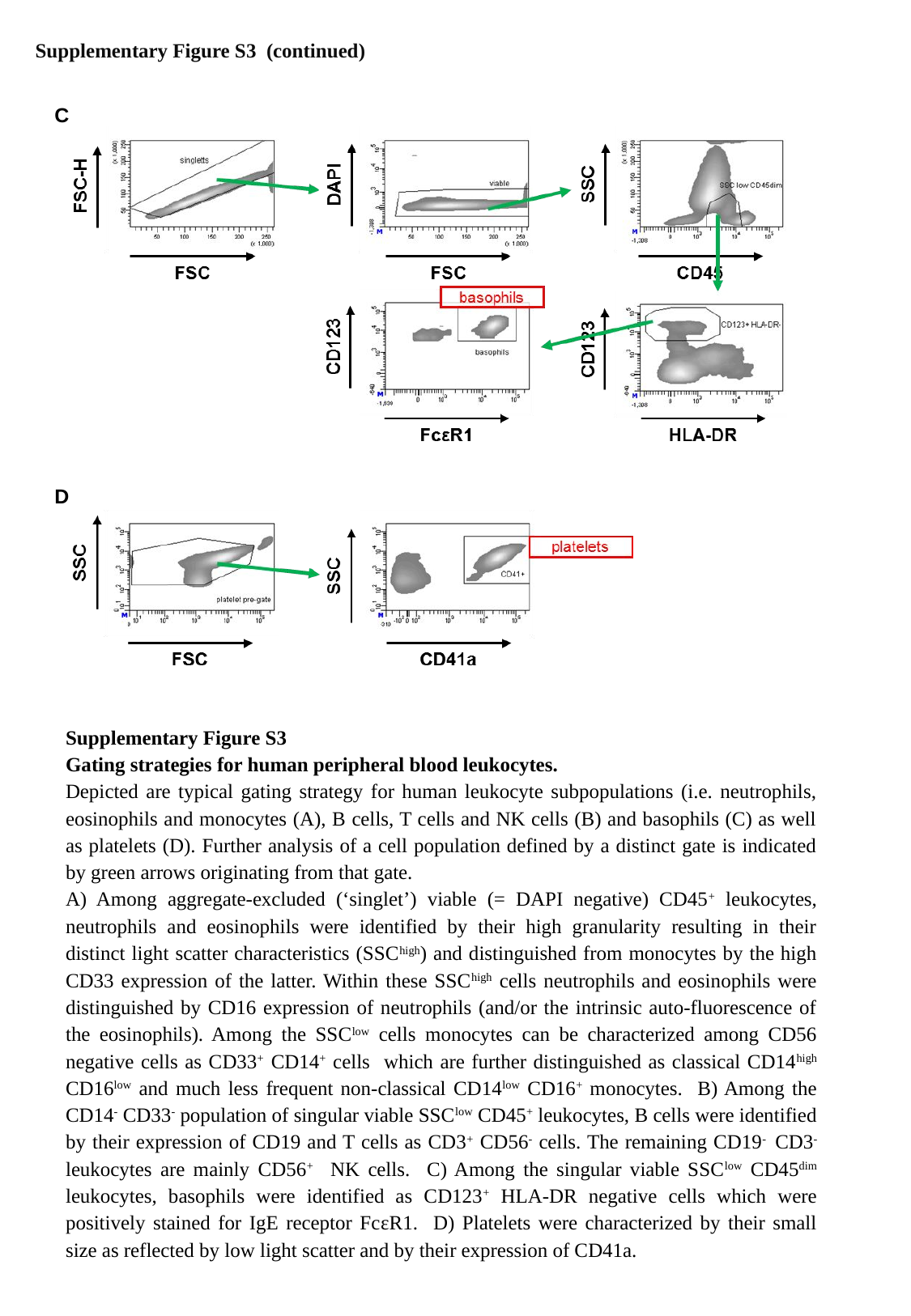

Supplementary Figure S3 (continued)
C
D
Supplementary Figure S3
Gating strategies for human peripheral blood leukocytes.
Depicted are typical gating strategy for human leukocyte subpopulations (i.e. neutrophils, eosinophils and monocytes (A), B cells, T cells and NK cells (B) and basophils (C) as well as platelets (D). Further analysis of a cell population defined by a distinct gate is indicated by green arrows originating from that gate.
A) Among aggregate-excluded (‘singlet’) viable (= DAPI negative) CD45+ leukocytes, neutrophils and eosinophils were identified by their high granularity resulting in their distinct light scatter characteristics (SSChigh) and distinguished from monocytes by the high CD33 expression of the latter. Within these SSChigh cells neutrophils and eosinophils were distinguished by CD16 expression of neutrophils (and/or the intrinsic auto-fluorescence of the eosinophils). Among the SSClow cells monocytes can be characterized among CD56 negative cells as CD33+ CD14+ cells which are further distinguished as classical CD14high CD16low and much less frequent non-classical CD14low CD16+ monocytes. B) Among the CD14- CD33- population of singular viable SSClow CD45+ leukocytes, B cells were identified by their expression of CD19 and T cells as CD3+ CD56- cells. The remaining CD19- CD3- leukocytes are mainly CD56+ NK cells. C) Among the singular viable SSClow CD45dim leukocytes, basophils were identified as CD123+ HLA-DR negative cells which were positively stained for IgE receptor FcεR1. D) Platelets were characterized by their small size as reflected by low light scatter and by their expression of CD41a.

## Slide 6
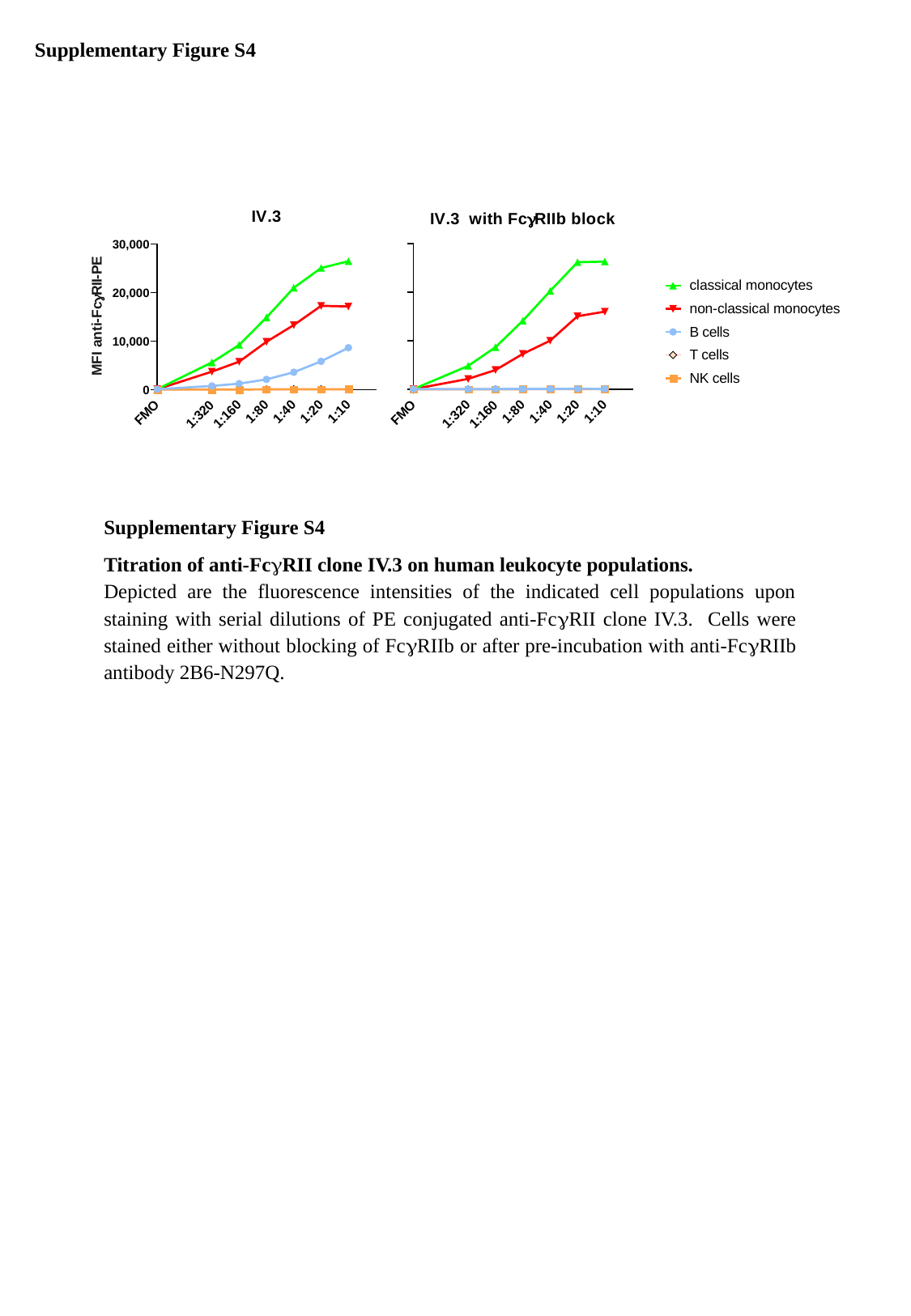

Supplementary Figure S4
Supplementary Figure S4
Titration of anti-FcγRII clone IV.3 on human leukocyte populations.
Depicted are the fluorescence intensities of the indicated cell populations upon staining with serial dilutions of PE conjugated anti-FcγRII clone IV.3. Cells were stained either without blocking of FcγRIIb or after pre-incubation with anti-FcγRIIb antibody 2B6-N297Q.

## Slide 7
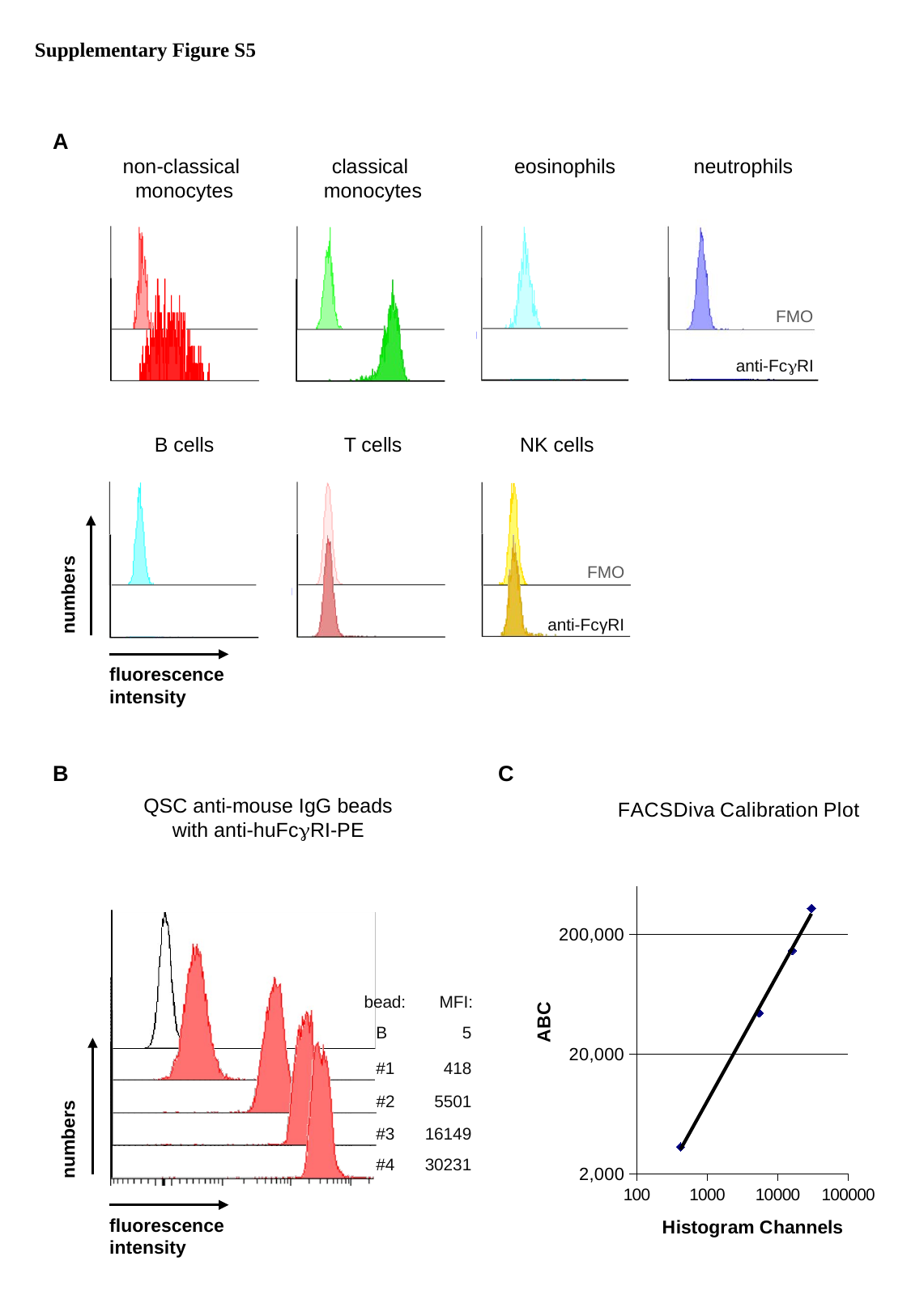

Supplementary Figure S5
A
non-classical
monocytes
classical
monocytes
eosinophils
neutrophils
FMO
anti-FcγRI
B cells
T cells
NK cells
FMO
numbers
anti-FcγRI
fluorescence
intensity
B
C
### Chart: FACSDiva Calibration Plot
| Category | |
|---|---|QSC anti-mouse IgG beads
with anti-huFcγRI-PE
MFI:
bead:
B	5
#1	418
#2	5501
#3	16149
#4	30231
numbers
fluorescence
intensity

## Slide 8
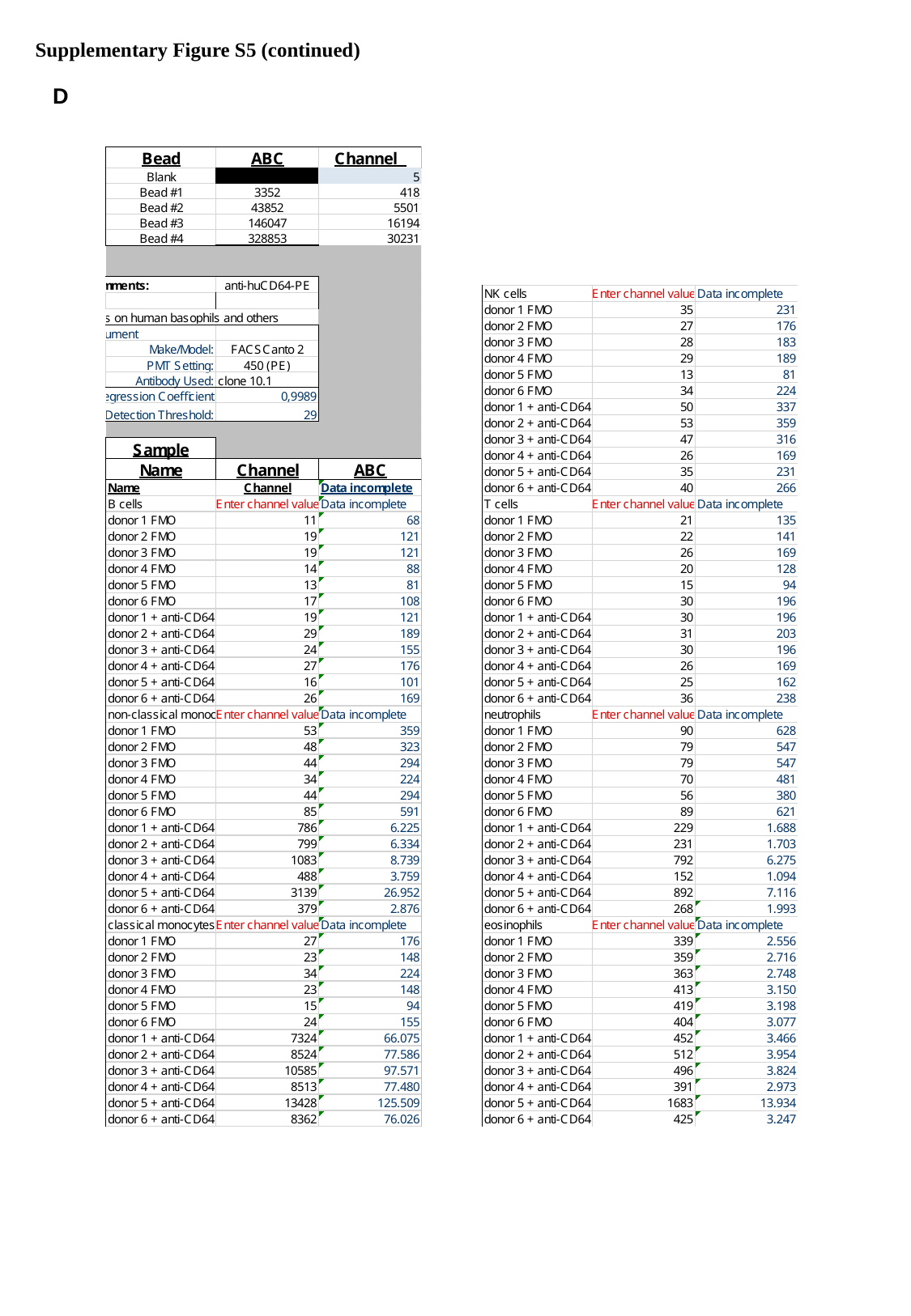

Supplementary Figure S5 (continued)
D

## Slide 9
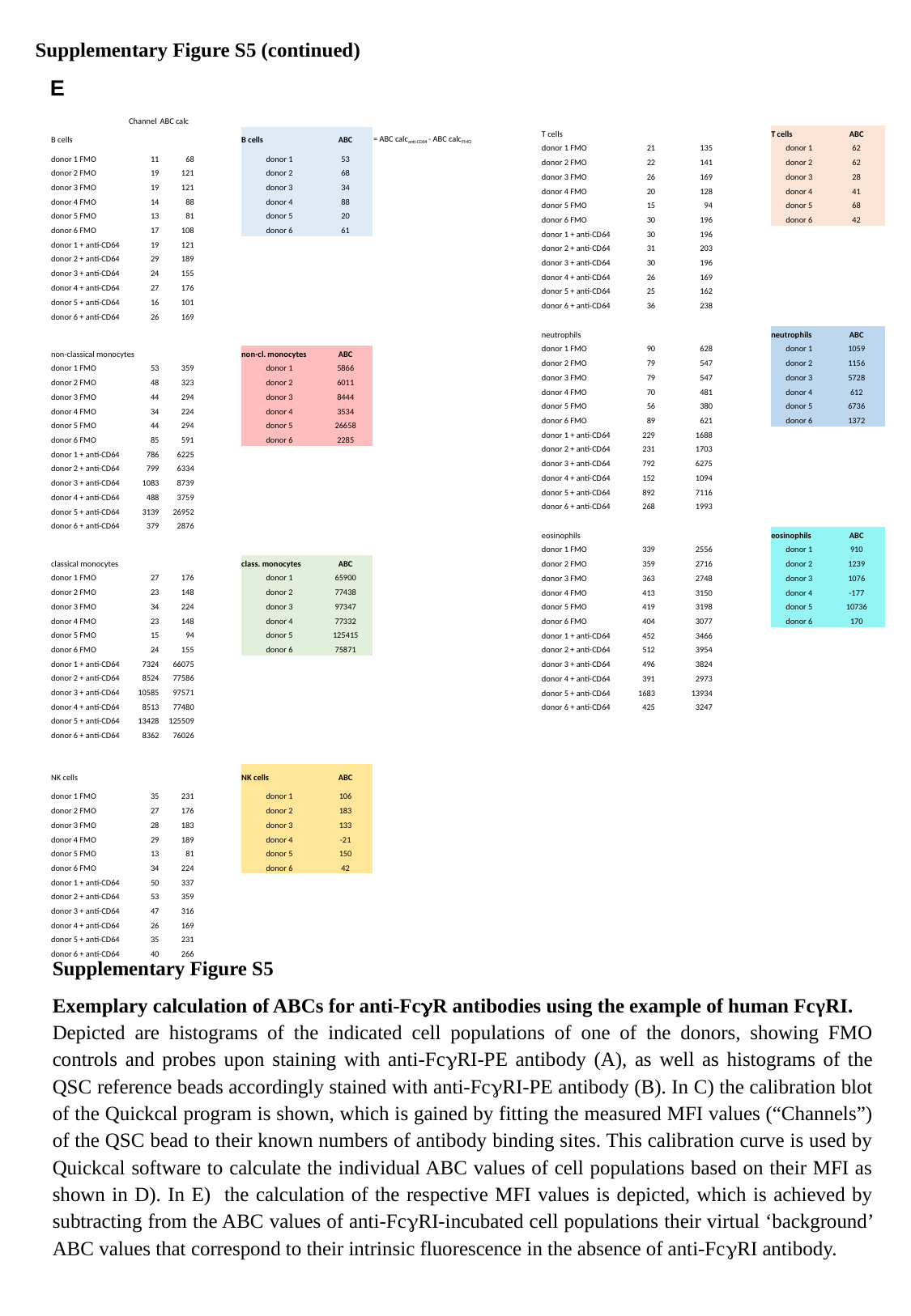

Supplementary Figure S5 (continued)
E
| | Channel | | ABC calc | | | | | | |
| --- | --- | --- | --- | --- | --- | --- | --- | --- | --- |
| B cells | | | | | B cells | ABC | = ABC calcanti-CD64 - ABC calcFMO | | |
| donor 1 FMO | 11 | | 68 | | donor 1 | 53 | | | |
| donor 2 FMO | 19 | | 121 | | donor 2 | 68 | | | |
| donor 3 FMO | 19 | | 121 | | donor 3 | 34 | | | |
| donor 4 FMO | 14 | | 88 | | donor 4 | 88 | | | |
| donor 5 FMO | 13 | | 81 | | donor 5 | 20 | | | |
| donor 6 FMO | 17 | | 108 | | donor 6 | 61 | | | |
| donor 1 + anti-CD64 | 19 | | 121 | | | | | | |
| donor 2 + anti-CD64 | 29 | | 189 | | | | | | |
| donor 3 + anti-CD64 | 24 | | 155 | | | | | | |
| donor 4 + anti-CD64 | 27 | | 176 | | | | | | |
| donor 5 + anti-CD64 | 16 | | 101 | | | | | | |
| donor 6 + anti-CD64 | 26 | | 169 | | | | | | |
| | | | | | | | | | |
| non-classical monocytes | | | | | non-cl. monocytes | ABC | | | |
| donor 1 FMO | 53 | | 359 | | donor 1 | 5866 | | | |
| donor 2 FMO | 48 | | 323 | | donor 2 | 6011 | | | |
| donor 3 FMO | 44 | | 294 | | donor 3 | 8444 | | | |
| donor 4 FMO | 34 | | 224 | | donor 4 | 3534 | | | |
| donor 5 FMO | 44 | | 294 | | donor 5 | 26658 | | | |
| donor 6 FMO | 85 | | 591 | | donor 6 | 2285 | | | |
| donor 1 + anti-CD64 | 786 | | 6225 | | | | | | |
| donor 2 + anti-CD64 | 799 | | 6334 | | | | | | |
| donor 3 + anti-CD64 | 1083 | | 8739 | | | | | | |
| donor 4 + anti-CD64 | 488 | | 3759 | | | | | | |
| donor 5 + anti-CD64 | 3139 | | 26952 | | | | | | |
| donor 6 + anti-CD64 | 379 | | 2876 | | | | | | |
| | | | | | | | | | |
| classical monocytes | | | | | class. monocytes | ABC | | | |
| donor 1 FMO | | 27 | 176 | | donor 1 | 65900 | | | |
| donor 2 FMO | | 23 | 148 | | donor 2 | 77438 | | | |
| donor 3 FMO | | 34 | 224 | | donor 3 | 97347 | | | |
| donor 4 FMO | | 23 | 148 | | donor 4 | 77332 | | | |
| donor 5 FMO | | 15 | 94 | | donor 5 | 125415 | | | |
| donor 6 FMO | | 24 | 155 | | donor 6 | 75871 | | | |
| donor 1 + anti-CD64 | | 7324 | 66075 | | | | | | |
| donor 2 + anti-CD64 | | 8524 | 77586 | | | | | | |
| donor 3 + anti-CD64 | | 10585 | 97571 | | | | | | |
| donor 4 + anti-CD64 | | 8513 | 77480 | | | | | | |
| donor 5 + anti-CD64 | | 13428 | 125509 | | | | | | |
| donor 6 + anti-CD64 | | 8362 | 76026 | | | | | | |
| | | | | | | | | | |
| NK cells | | | | | NK cells | ABC | | | |
| donor 1 FMO | | 35 | 231 | | donor 1 | 106 | | | |
| donor 2 FMO | | 27 | 176 | | donor 2 | 183 | | | |
| donor 3 FMO | | 28 | 183 | | donor 3 | 133 | | | |
| donor 4 FMO | | 29 | 189 | | donor 4 | -21 | | | |
| donor 5 FMO | | 13 | 81 | | donor 5 | 150 | | | |
| donor 6 FMO | | 34 | 224 | | donor 6 | 42 | | | |
| donor 1 + anti-CD64 | | 50 | 337 | | | | | | |
| donor 2 + anti-CD64 | | 53 | 359 | | | | | | |
| donor 3 + anti-CD64 | | 47 | 316 | | | | | | |
| donor 4 + anti-CD64 | | 26 | 169 | | | | | | |
| donor 5 + anti-CD64 | | 35 | 231 | | | | | | |
| donor 6 + anti-CD64 | | 40 | 266 | | | | | | |
| T cells | | | | T cells | ABC |
| --- | --- | --- | --- | --- | --- |
| donor 1 FMO | 21 | 135 | | donor 1 | 62 |
| donor 2 FMO | 22 | 141 | | donor 2 | 62 |
| donor 3 FMO | 26 | 169 | | donor 3 | 28 |
| donor 4 FMO | 20 | 128 | | donor 4 | 41 |
| donor 5 FMO | 15 | 94 | | donor 5 | 68 |
| donor 6 FMO | 30 | 196 | | donor 6 | 42 |
| donor 1 + anti-CD64 | 30 | 196 | | | |
| donor 2 + anti-CD64 | 31 | 203 | | | |
| donor 3 + anti-CD64 | 30 | 196 | | | |
| donor 4 + anti-CD64 | 26 | 169 | | | |
| donor 5 + anti-CD64 | 25 | 162 | | | |
| donor 6 + anti-CD64 | 36 | 238 | | | |
| | | | | | |
| neutrophils | | | | neutrophils | ABC |
| donor 1 FMO | 90 | 628 | | donor 1 | 1059 |
| donor 2 FMO | 79 | 547 | | donor 2 | 1156 |
| donor 3 FMO | 79 | 547 | | donor 3 | 5728 |
| donor 4 FMO | 70 | 481 | | donor 4 | 612 |
| donor 5 FMO | 56 | 380 | | donor 5 | 6736 |
| donor 6 FMO | 89 | 621 | | donor 6 | 1372 |
| donor 1 + anti-CD64 | 229 | 1688 | | | |
| donor 2 + anti-CD64 | 231 | 1703 | | | |
| donor 3 + anti-CD64 | 792 | 6275 | | | |
| donor 4 + anti-CD64 | 152 | 1094 | | | |
| donor 5 + anti-CD64 | 892 | 7116 | | | |
| donor 6 + anti-CD64 | 268 | 1993 | | | |
| | | | | | |
| eosinophils | | | | eosinophils | ABC |
| donor 1 FMO | 339 | 2556 | | donor 1 | 910 |
| donor 2 FMO | 359 | 2716 | | donor 2 | 1239 |
| donor 3 FMO | 363 | 2748 | | donor 3 | 1076 |
| donor 4 FMO | 413 | 3150 | | donor 4 | -177 |
| donor 5 FMO | 419 | 3198 | | donor 5 | 10736 |
| donor 6 FMO | 404 | 3077 | | donor 6 | 170 |
| donor 1 + anti-CD64 | 452 | 3466 | | | |
| donor 2 + anti-CD64 | 512 | 3954 | | | |
| donor 3 + anti-CD64 | 496 | 3824 | | | |
| donor 4 + anti-CD64 | 391 | 2973 | | | |
| donor 5 + anti-CD64 | 1683 | 13934 | | | |
| donor 6 + anti-CD64 | 425 | 3247 | | | |
Supplementary Figure S5
Exemplary calculation of ABCs for anti-FcγR antibodies using the example of human FcγRI.
Depicted are histograms of the indicated cell populations of one of the donors, showing FMO controls and probes upon staining with anti-FcγRI-PE antibody (A), as well as histograms of the QSC reference beads accordingly stained with anti-FcγRI-PE antibody (B). In C) the calibration blot of the Quickcal program is shown, which is gained by fitting the measured MFI values (“Channels”) of the QSC bead to their known numbers of antibody binding sites. This calibration curve is used by Quickcal software to calculate the individual ABC values of cell populations based on their MFI as shown in D). In E) the calculation of the respective MFI values is depicted, which is achieved by subtracting from the ABC values of anti-FcγRI-incubated cell populations their virtual ‘background’ ABC values that correspond to their intrinsic fluorescence in the absence of anti-FcγRI antibody.

## Slide 10
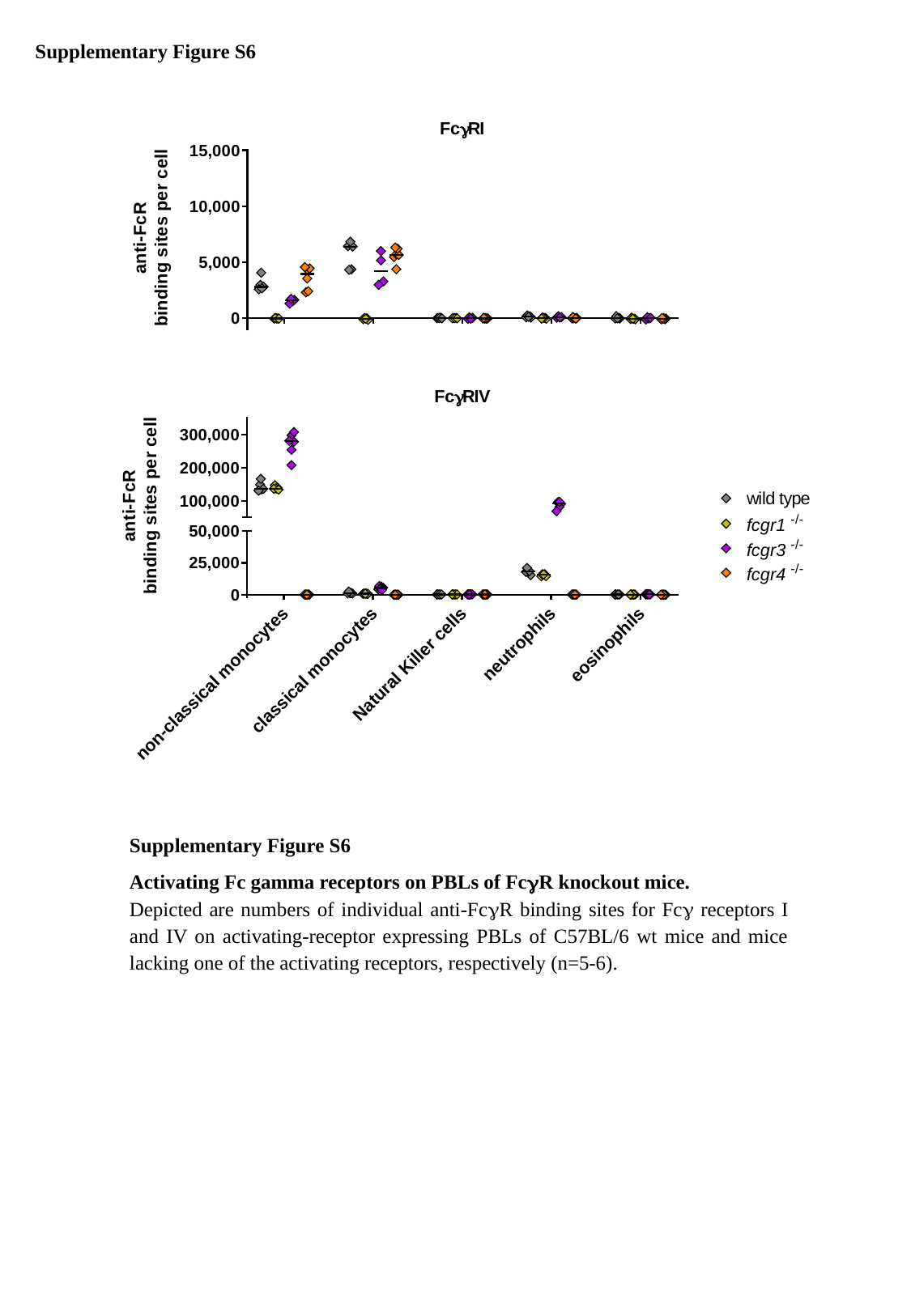

Supplementary Figure S6
Supplementary Figure S6
Activating Fc gamma receptors on PBLs of FcγR knockout mice.
Depicted are numbers of individual anti-FcγR binding sites for Fcγ receptors I and IV on activating-receptor expressing PBLs of C57BL/6 wt mice and mice lacking one of the activating receptors, respectively (n=5-6).

## Slide 11
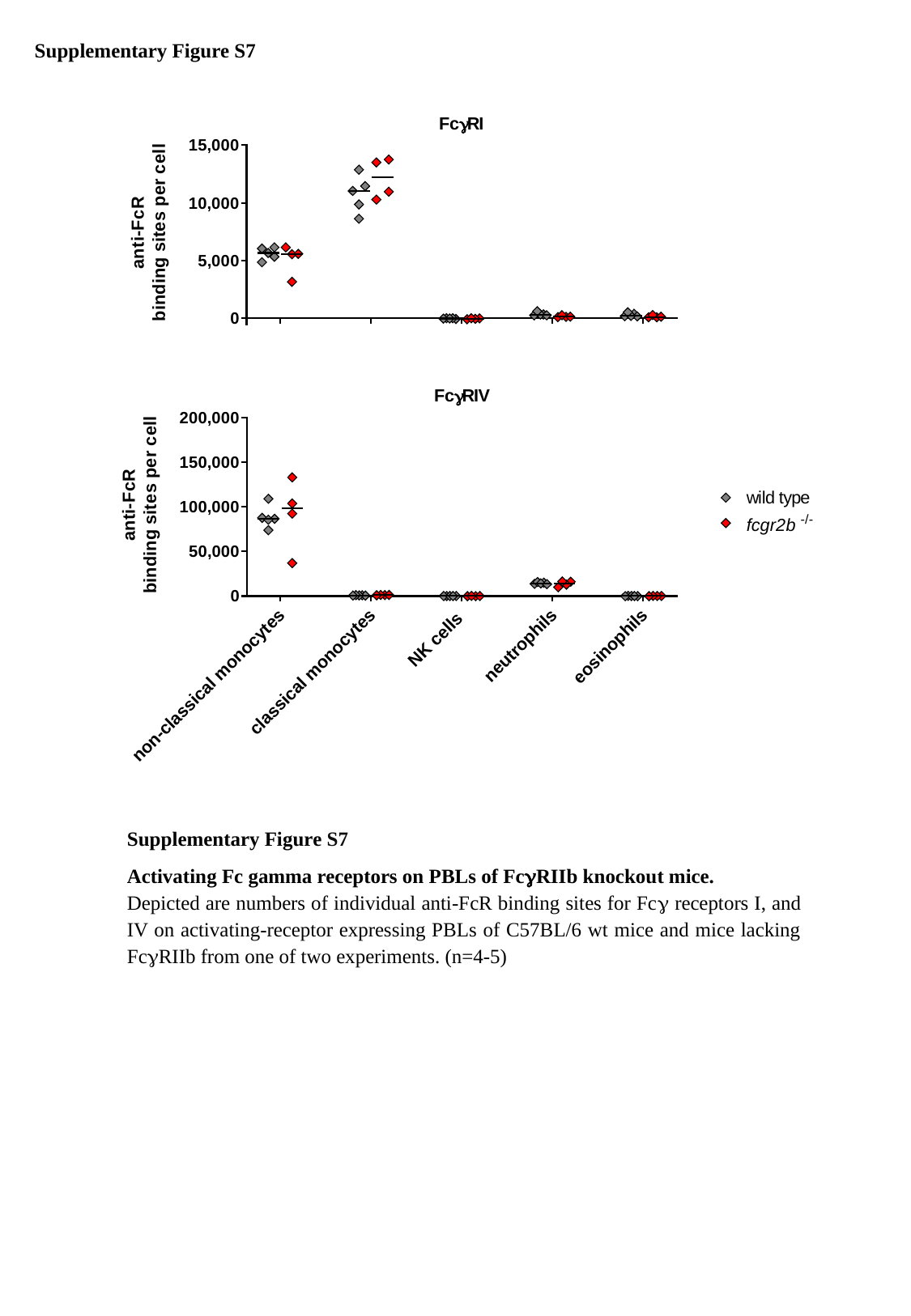

Supplementary Figure S7
Supplementary Figure S7
Activating Fc gamma receptors on PBLs of FcγRIIb knockout mice.
Depicted are numbers of individual anti-FcR binding sites for Fcγ receptors I, and IV on activating-receptor expressing PBLs of C57BL/6 wt mice and mice lacking FcγRIIb from one of two experiments. (n=4-5)

## Slide 12
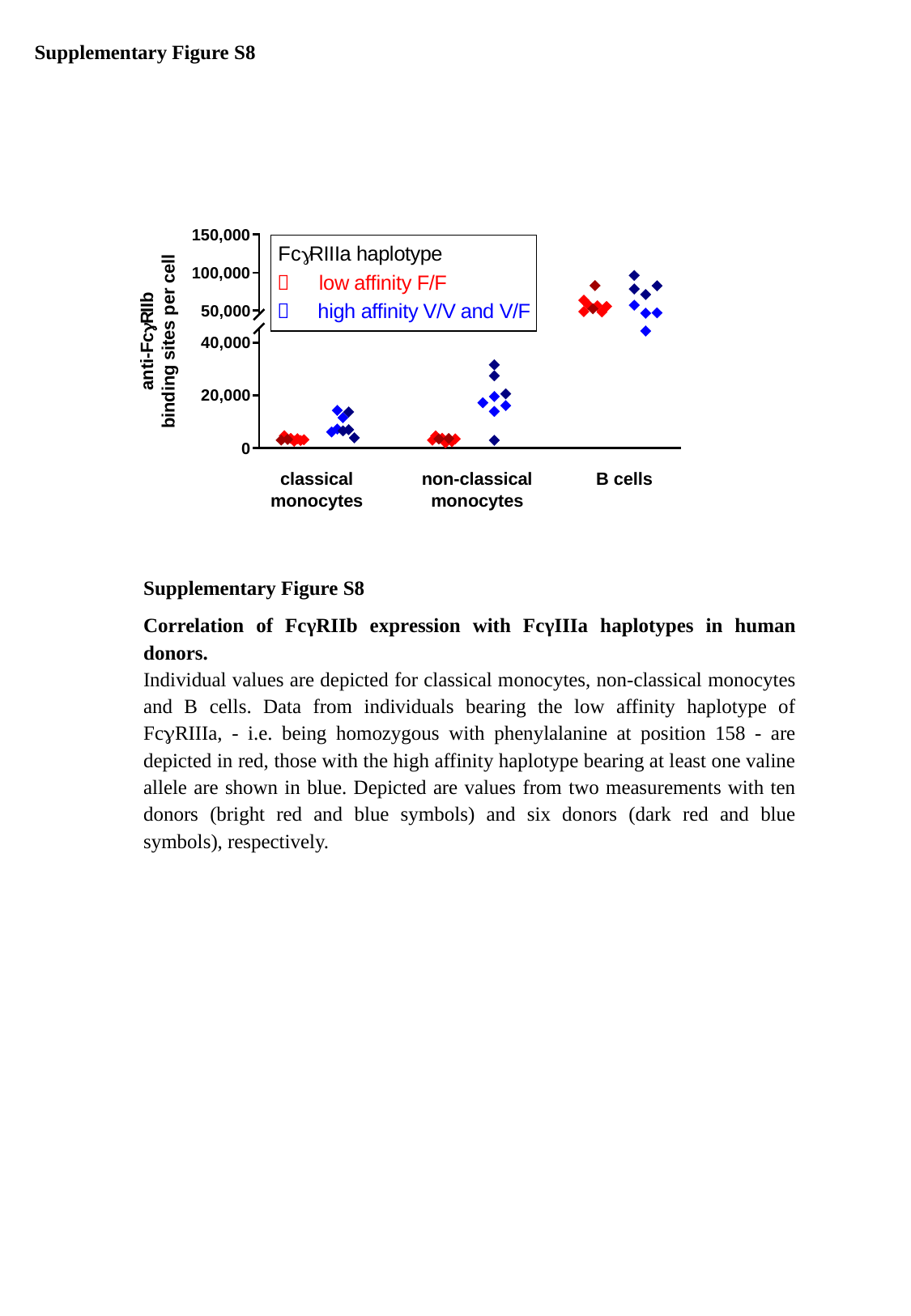

Supplementary Figure S8
classical
monocytes
non-classical
monocytes
B cells
Supplementary Figure S8
Correlation of FcγRIIb expression with FcγIIIa haplotypes in human donors.
Individual values are depicted for classical monocytes, non-classical monocytes and B cells. Data from individuals bearing the low affinity haplotype of FcγRIIIa, - i.e. being homozygous with phenylalanine at position 158 - are depicted in red, those with the high affinity haplotype bearing at least one valine allele are shown in blue. Depicted are values from two measurements with ten donors (bright red and blue symbols) and six donors (dark red and blue symbols), respectively.

## Slide 13
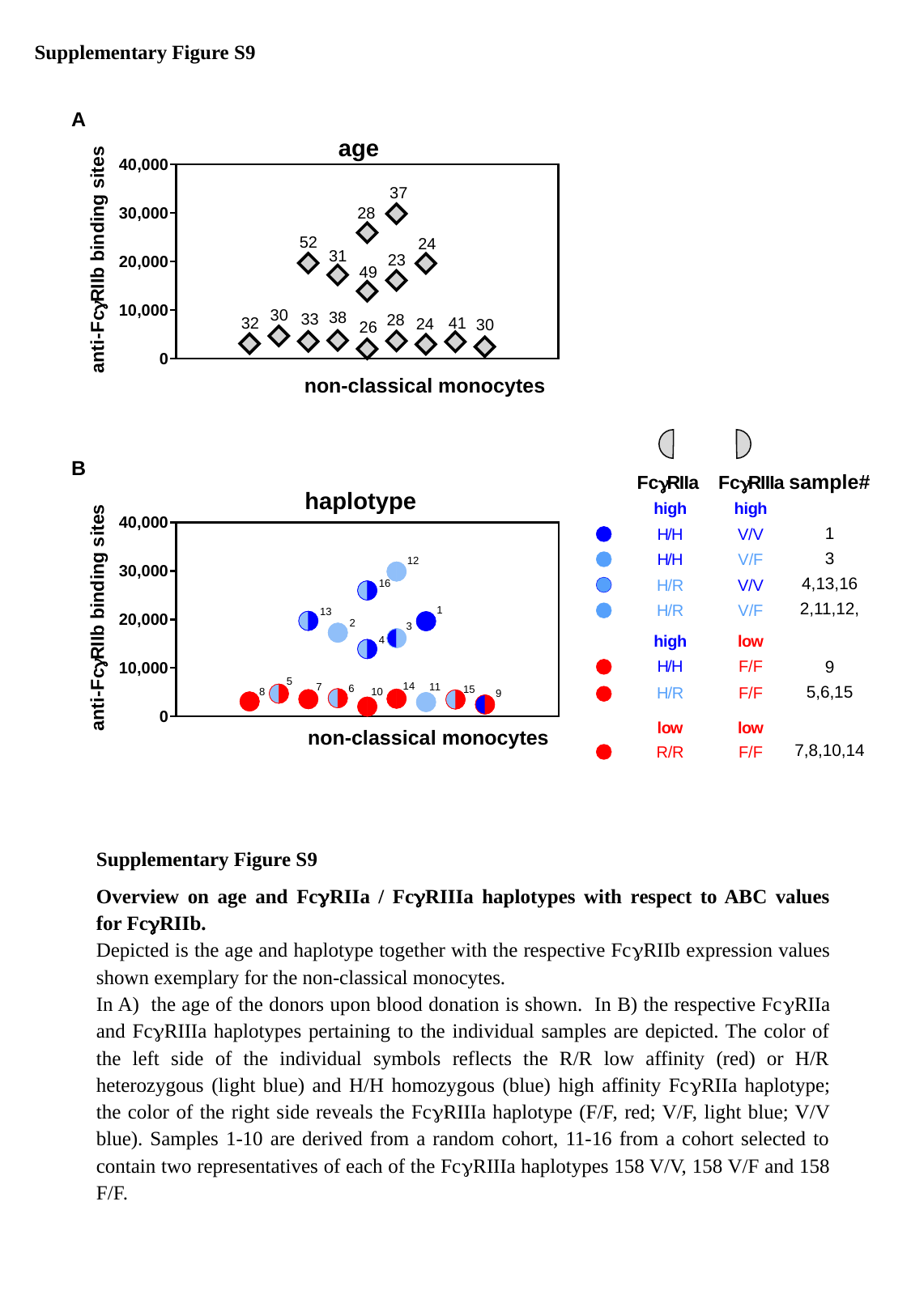

Supplementary Figure S9
A
age
non-classical monocytes
B
sample#
1
3
4,13,16
2,11,12,
9
5,6,15
7,8,10,14
haplotype
non-classical monocytes
Supplementary Figure S9
Overview on age and FcγRIIa / FcγRIIIa haplotypes with respect to ABC values
for FcγRIIb.
Depicted is the age and haplotype together with the respective FcγRIIb expression values shown exemplary for the non-classical monocytes.
In A) the age of the donors upon blood donation is shown. In B) the respective FcγRIIa and FcγRIIIa haplotypes pertaining to the individual samples are depicted. The color of the left side of the individual symbols reflects the R/R low affinity (red) or H/R heterozygous (light blue) and H/H homozygous (blue) high affinity FcγRIIa haplotype; the color of the right side reveals the FcγRIIIa haplotype (F/F, red; V/F, light blue; V/V blue). Samples 1-10 are derived from a random cohort, 11-16 from a cohort selected to contain two representatives of each of the FcγRIIIa haplotypes 158 V/V, 158 V/F and 158 F/F.

## Slide 14
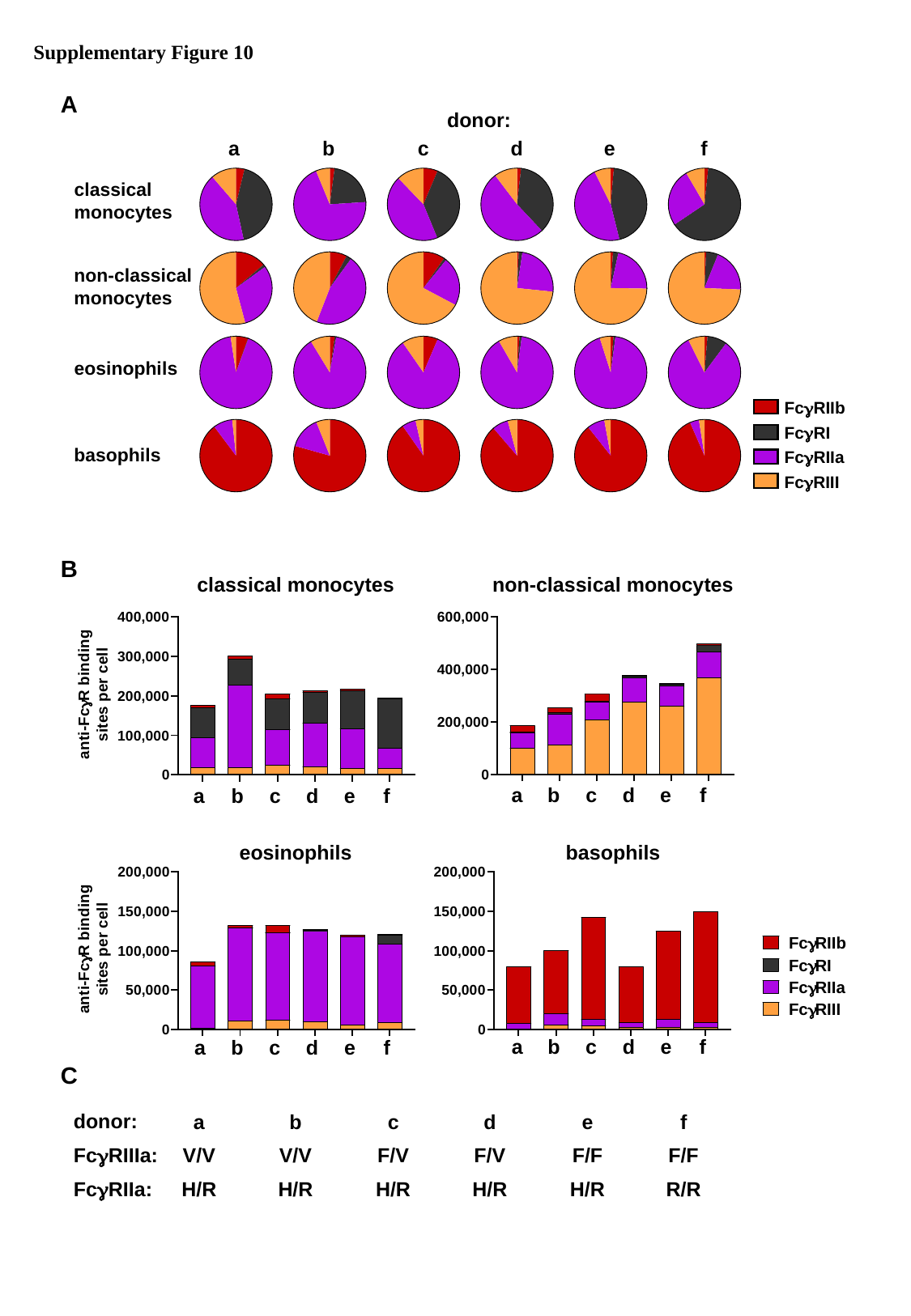

Supplementary Figure 10
A
donor:
a
b
c
d
e
f
classical
monocytes
non-classical
monocytes
eosinophils
FcγRIIb
FcγRI
basophils
FcγRIIa
FcγRIII
B
classical monocytes
non-classical monocytes
a
b
c
d
e
f
a
b
c
d
e
f
eosinophils
basophils
a
b
c
d
e
f
a
b
c
d
e
f
C
donor:
FcγRIIIa:
FcγRIIa:
a
V/V
H/R
b
V/V
H/R
c
F/V
H/R
d
F/V
H/R
e
F/F
H/R
f
F/F
R/R

## Slide 15
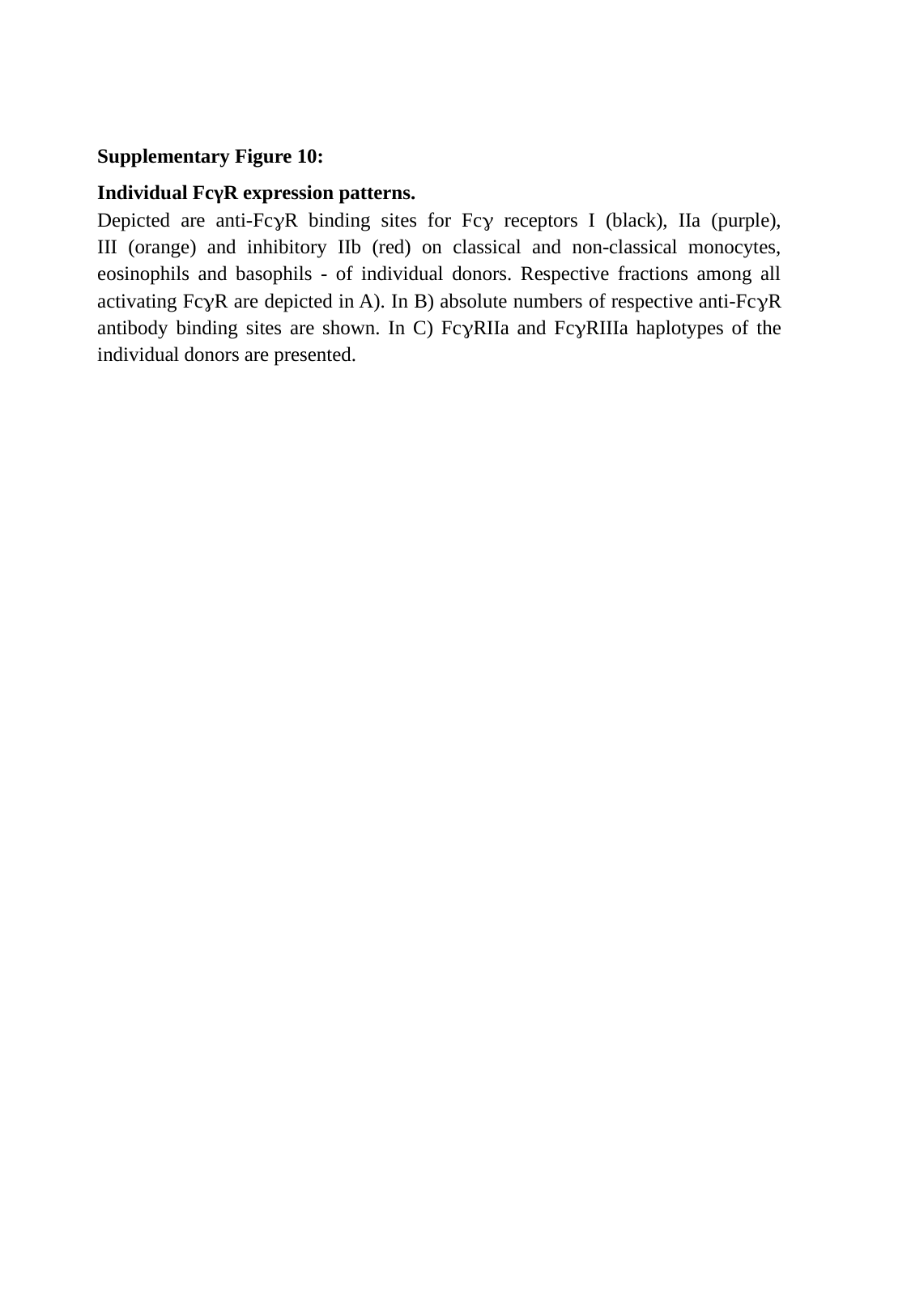

Supplementary Figure 10:
Individual FcγR expression patterns.
Depicted are anti-FcγR binding sites for Fcγ receptors I (black), IIa (purple),
III (orange) and inhibitory IIb (red) on classical and non-classical monocytes, eosinophils and basophils - of individual donors. Respective fractions among all activating FcγR are depicted in A). In B) absolute numbers of respective anti-FcγR antibody binding sites are shown. In C) FcγRIIa and FcγRIIIa haplotypes of the individual donors are presented.
